# Blocking Synthesis of the Variant Surface Glycoprotein Coat in *Trypanosoma brucei* Leads to an Increase in Macrophage Phagocytosis Due to Reduced Clearance of Surface Coat Antibodies

**DOI:** 10.1371/journal.ppat.1006023

**Published:** 2016-11-28

**Authors:** Jackie L. Y. Cheung, Nadina V. Wand, Cher-Pheng Ooi, Sophie Ridewood, Richard J. Wheeler, Gloria Rudenko

**Affiliations:** 1 Department of Life Sciences, Sir Alexander Fleming Building, Imperial College London, South Kensington, London, United Kingdom; 2 Department of Pathology, Sir William Dunn School of Pathology, University of Oxford, Oxford, United Kingdom; University of California, Los Angeles, UNITED STATES

## Abstract

The extracellular bloodstream form parasite *Trypanosoma brucei* is supremely adapted to escape the host innate and adaptive immune system. Evasion is mediated through an antigenically variable Variant Surface Glycoprotein (VSG) coat, which is recycled at extraordinarily high rates. Blocking VSG synthesis triggers a precytokinesis arrest where stalled cells persist for days *in vitro* with superficially intact VSG coats, but are rapidly cleared within hours in mice. We therefore investigated the role of VSG synthesis in trypanosome phagocytosis by activated mouse macrophages. *T*. *brucei* normally effectively evades macrophages, and induction of *VSG* RNAi resulted in little change in phagocytosis of the arrested cells. Halting VSG synthesis resulted in stalled cells which swam directionally rather than tumbling, with a significant increase in swim velocity. This is possibly a consequence of increased rigidity of the cells due to a restricted surface coat in the absence of VSG synthesis. However if *VSG* RNAi was induced in the presence of anti-VSG221 antibodies, phagocytosis increased significantly. Blocking VSG synthesis resulted in reduced clearance of anti-VSG antibodies from the trypanosome surface, possibly as a consequence of the changed motility. This was particularly marked in cells in the G2/ M cell cycle stage, where the half-life of anti-VSG antibody increased from 39.3 ± 4.2 seconds to 99.2 ± 15.9 seconds after induction of *VSG* RNAi. The rates of internalisation of bulk surface VSG, or endocytic markers like transferrin, tomato lectin or dextran were not significantly affected by the VSG synthesis block. Efficient elimination of anti-VSG-antibody complexes from the trypanosome cell surface is therefore essential for trypanosome evasion of macrophages. These experiments highlight the essentiality of high rates of VSG recycling for the rapid removal of host opsonins from the parasite surface, and identify this process as a key parasite virulence factor during a chronic infection.

## Author Summary

Bloodstream form African trypanosomes infect the mammalian bloodstream as extracellular parasites, where they excel at escaping the immune system including elimination by macrophages. Key for survival is a dense Variant Surface Glycoprotein (VSG) coat which is antigenically varied, and which is recycled from the cell surface at very high rates. VSG is essential, and blocking its synthesis produces cells which do not grow, but persist for days. Here, we investigated the role of the VSG coat in trypanosome escape from macrophages. Arresting VSG synthesis resulted in trypanosomes with changed motility, which were not phagocytosed at higher rates. However, phagocytosis was dramatically increased if a VSG synthesis block was induced before the trypanosomes were coated with anti-VSG antibodies. Pre-treatment with host complement had a more moderate effect on phagocytosis. We showed that the arrested cells had reduced rates of clearance of anti-VSG antibody from their surface, making them a target for recognition by macrophages. These experiments highlight the critical importance of high rates of VSG internalisation for the rapid removal of host immune molecules including antibodies and complement from the trypanosome cell surface. This allows trypanosomes to effectively evade macrophages during a chronic infection.

## Introduction

The African trypanosome *Trypanosoma brucei* is a unicellular parasite uniquely adapted to parasitize the mammalian bloodstream after inoculation by tsetse flies. Here, *T*. *brucei* establishes chronic infections leading to devastating diseases such as Human African Trypanosomiasis (HAT) or ‘nagana’ in livestock. *T*. *brucei* can also infect a broad range of African mammals, which tolerate low grades of trypanosome infection and serve as reservoirs for disease [1, 2]. Within the blood, as an extracellular pathogen *T*. *brucei* is confronted with continuous attack from the innate and adaptive arms of the host immune system. These include the complement system, antibodies and phagocytic cells such as macrophages. An essential trypanosome survival feature within this hostile environment, is a protective coat of Variant Surface Glycoprotein (VSG) [3, 4].

VSG is key for evasion of the adaptive immune response, and extraordinarily sophisticated antigenic variation of VSG allows the trypanosome to switch stochastically between different VSG variants during the course of a chronic infection. Individual trypanosomes express one VSG out of a vast repertoire of up to 2000 *VSG* genes and pseudogenes from one of about 15 telomeric *VSG* expression site (ES) transcription units [5, 6]. Switching the active *VSG* can involve transcriptional switching between different ESs, or DNA rearrangements slotting new *VSG*s (or *VSG* segments) into the active ES [7, 8]. In addition to its role in immune evasion through VSG switching, the very dense VSG surface presumably prevents the host complement system from rapidly forming lytic pores in the parasite in the form of membrane attack complexes (MAC).

VSG therefore forms a protective barrier with highly intriguing properties. VSG is the most abundant protein in bloodstream form *T*. *brucei* (~10^7^ molecules) and the VSG coat is an extremely dense layer of tightly packed VSG molecules [4, 9, 10]. The VSG coat has been modelled with VSG approaching maximal densities [11]. However, it is still unclear whether VSG is indeed normally fully saturated at the cell surface [12].

VSG is essential even *in vitro*, and blocking its synthesis does not lead to a major reduction in VSG coat density [13]. Instead, a unique cell cycle checkpoint is triggered, leading to trypanosomes which have stalled at a precise precytokinesis stage. Concurrently, a global translation arrest is induced, resulting in stalled cells which do not grow, again preventing catastrophic dilution of the protective VSG surface [14]. Despite its dense nature, the VSG layer forms a semi-permeable barrier blocking deposition of host complement proteins and antibodies on the trypanosome surface membrane [9, 15–17], yet allowing smaller nutrient molecules to reach surface receptors [18]. As VSG proteins are attached to the cell surface through glycosylphosphatidylinositol (GPI) anchors, VSG molecules can move freely on the cell surface forming an unusually fluid protective surface [19].

This highly fluid nature of the VSG surface coat facilitates another unusual *T*. *brucei* feature, and that is extremely high rates of VSG recycling [20]. Trypanosomes compartmentalise endocytosis and exocytosis to a specialised invagination at the base of the flagellum known as the flagellar pocket [18, 21]. *T*. *brucei* has spectacularly high rates of recycling of VSG, and it is estimated that the entire surface complement of VSG is internalised once every 12 minutes [20]. Within the cell, a stringent quality control mechanism monitors which VSGs are returned back to the cell surface. Defective VSGs (and presumably VSGs with covalently bound complement proteins) are targeted for degradation [22]. Internalised VSG-antibody complexes are also prevented from returning back to the cell surface [23].

This high rate of VSG recycling in addition to quality control mechanisms, are key trypanosome virulence factors allowing trypanosomes to multiply unimpeded in increasing titres of anti-VSG antibody [17, 24]. It is only when anti-VSG titres rise above a critical level that recognised trypanosomes are overwhelmed, and parasites expressing the predominant VSG variant are exterminated. These extraordinarily high rates of VSG recycling therefore function as an unusual ‘coat-cleaning’ machine, resulting in the rapid removal of lethal immune complexes from the cell surface [24]. Motility of the trypanosome appears to facilitate this removal. The swimming trypanosome is thought to generate hydrodynamic forces, which preferentially push VSG-antibody complexes back through the VSG layer towards the flagellar pocket where they are endocytosed [24].

In addition to evading complement and antibodies, bloodstream form African trypanosomes must evade phagocytes including macrophages [25, 26]. Although some aspects surrounding the immunology of macrophage activation during a *T*. *brucei* infection have been investigated [27–30], little is known about how phagocytosis of trypanosomes by macrophages occurs mechanistically. We have shown earlier that blocking VSG synthesis *in vitro* leads to stalled trypanosomes which persist for days [13]. However, if *VSG* RNAi is induced *in vivo* in infected mice, trypanosomes are very rapidly cleared within hours. We have no evidence that blocking VSG synthesis results in increased trypanosome sensitivity to the alternative pathway of complement mediated lysis.

In order to understand this rapid trypanosome clearance *in vivo*, we therefore investigated the effect of blocking VSG synthesis on trypanosome phagocytosis by macrophages. Macrophages can be co-cultured with trypanosomes *in vitro*, providing a powerful experimental system for investigating the role of phagocytes during a trypanosome infection. Here, we found that although blocking VSG synthesis resulted in altered motility of the stalled cells, there was no significant increase in phagocytosis by mouse macrophages. However phagocytosis of the arrested trypanosomes was significantly increased in the presence of anti-VSG antibodies. Concurrently, we observed a significant decrease in rates of clearance of anti-VSG complexes from the trypanosome surface when VSG synthesis was blocked. These experiments show that an experimental perturbation resulting in reduced clearance of anti-VSG antibodies directly inhibits the ability of the trypanosome to evade phagocytosis. These experiments highlight the importance of both high rates of VSG synthesis and clearance of anti-VSG antibodies for trypanosome evasion from the multiple arms of the host immune system including phagocytes.

## Results

### Blocking VSG synthesis leads to increased phagocytosis of trypanosomes in the presence of anti-VSG antibodies

The induction of a VSG synthesis block in *T*. *brucei in vitro* leads to an abrupt inhibition of growth within one cell cycle (8 hours), and a precise pre-cytokinesis cell cycle arrest [13]. VSG is highly essential for bloodstream form *T*. *brucei in vivo*, and blocking its synthesis results in rapid elimination of a trypanosome infection in mice within hours. In order to understand how this rapid clearance *in vivo* is mediated, we investigated the effect of inhibiting VSG synthesis on trypanosome phagocytosis by mouse macrophages *in vitro*. To study this, we co-cultivated *T*. *brucei* 221VG1.1 in the presence of activated mouse RAW264.7 macrophages for one hour at 37°C. In order to trigger classical activation, macrophages were first stimulated with mouse IFN-γ (interferon gamma) and LPS (Lipopolysaccharides from *E*. *coli*) for 24 hours prior to co-cultivation with trypanosomes [31, 32].

**Fig 1:**
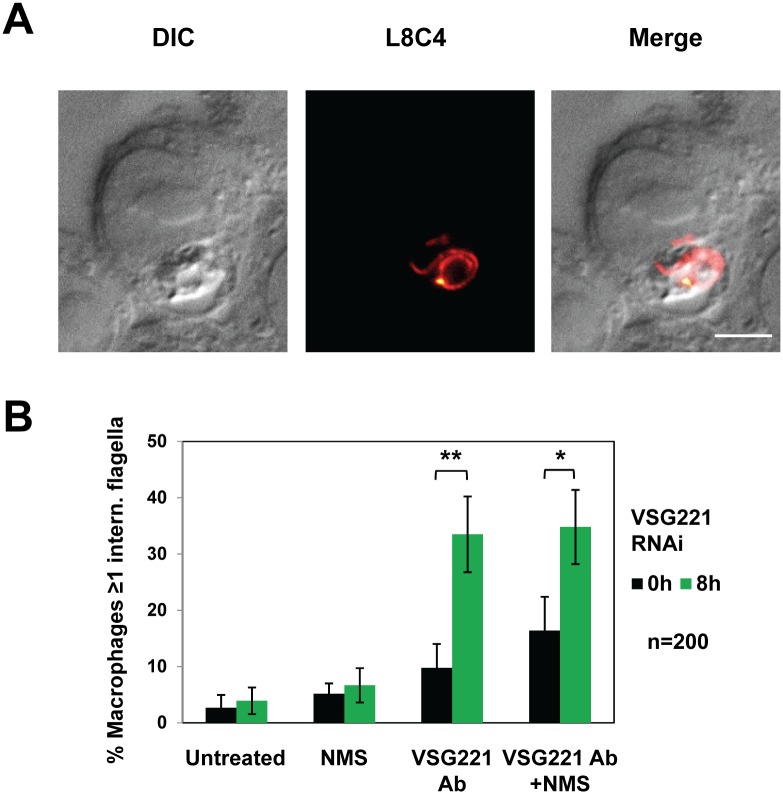
Inhibition of VSG synthesis in *T*. *brucei* leads to an increase in phagocytosis by macrophages in the presence of anti-VSG221 antibodies. **(A)** Phagocytosis of *T*. *brucei* by mouse RAW264.7 macrophages. The cells are visualised by differential interference contrast (DIC) or after immunofluorescence microscopy using a monoclonal antibody against the *T*. *brucei* paraflagellar rod protein PFR2 (L8C4). This allows visualisation of *T*. *brucei* after phagocytosis by the macrophage. Scale bar: 5 μm. **(B)** Quantitation of the percentage of macrophages with one or more internalised *T*. *brucei* flagella. VSG synthesis was blocked by the induction of *VSG221* RNAi in *T*. *brucei* 221VG1.1 for 0 or 8 hours (h). Parasites were then either left untreated, or were opsonised with normal mouse serum (NMS), polyclonal anti-VSG221 antibody (Ab) or both NMS and anti-VSG221 antibody. Phagocytosis of *T*. *brucei* was quantified by immunofluorescence microscopy, and was found to be significantly increased in the presence of anti-VSG antibodies and NMS (single asterisk), or after induction of a VSG synthesis block in the presence of anti-VSG antibodies (asterisks over brackets). The results are the mean from three independent experiments (n = 200) with the standard deviation indicated with error bars. Statistical analysis was performed using the Student’s t-test **P<0.001, *P<0.05.

The *T*. *brucei* 221VG1.1 trypanosome line contains *eGFP* and a puromycin resistance gene inserted in the active *VSG221* expression site, and a construct allowing tetracycline inducible expression of *VSG221* RNAi [13]. VSG synthesis was blocked by the induction of *VSG221* RNAi with tetracycline for eight hours, which is the earliest time point that produces a full cell-cycle arrest. Trypanosome phagocytosis was analysed by immunofluorescence microscopy, allowing visualisation of *T*. *brucei* flagella even after internalisation within the mouse macrophage using a monoclonal antibody (L8C4) against the *T*. *brucei* paraflagellar rod protein 2 (PFR2) [33] (Fig 1A).

As expected for a bloodstream pathogen, bloodstream form *T*. *brucei* normally escapes significant phagocytosis by macrophages. When macrophages were co-cultivated with bloodstream form *T*. *brucei*, only 2.7 ± 2.3% of the macrophages showed evidence of internalised parasites (Fig 1B). Host molecules including complement C3b or IgG or IgM antibodies adhere to microbial invaders, thereby facilitating phagocytosis of the opsonised pathogens [25, 34]. Macrophages adhere to these host protein coated pathogens through specific surface receptors recognising these opsonins [26, 35]. We therefore investigated phagocytosis of *T*. *brucei* after pre-incubation with either complement proteins or anti-VSG antibodies. The complement used in these experiments was present in normal mouse serum (NMS) from the CD-1 mouse strain. Unlike many laboratory mouse strains, CD-1 mice have functional complement proteins of the terminal complement pathway, and have been shown to have functional serum complement activity of the classical, lectin and alternative pathways at the level of C9 activation [36]. To verify that the normal mouse serum from our CD-1 mice was indeed complement active, we showed activity of the classical pathway (requiring both antibodies and complement). After opsonisation for 90 minutes with both NMS and anti-VSG antibodies, there was moderate although statistically significant lysis of *T*. *brucei* after the induction of *VSG221* RNAi for eight hours (*P<0.05) (S1 Fig). The anti-VSG221 antibody used in our experiments was a rabbit polyclonal antibody (1:5000 dilution), which was not in excess in our assays, as shown in S2 Fig.

In order to investigate the role of host opsonins on phagocytosis of *T*. *brucei*, we incubated *T*. *brucei* with NMS from CD1 mice, anti-VSG221 antibody, or both NMS and anti-VSG221 antibodies before co-culturing the trypanosomes with activated mouse RAW264.7 macrophages (Fig 1B). Prior incubation of trypanosomes with NMS did not lead to a significant increase in macrophages with phagocytosed parasites (up to 5.2 ± 1.9%). A possible increase in phagocytosis was observed if trypanosomes were first incubated with anti-VSG221 antibodies (up to 9.8 ± 4.3%), however this increase (up 16.4 ± 6.0%) was only statistically significant (*P<0.05) if normal mouse serum was added together with anti-VSG221 antibodies. This established the importance of host opsonins for phagocytosis of *T*. *brucei*. In addition, these data indicate the importance of the classical pathway of complement activation, whereby dedicated host complement proteins adhere to host antibodies coating microorganisms.

We next investigated the role of the VSG coat in trypanosome recognition and phagocytosis by macrophages. The induction of a VSG synthesis block leads to an arrest in trypanosome growth, with the majority of the stalled population (> 65%) consisting of bi-flagellated cells arrested precytokinesis [13]. Surprisingly, we found that blocking VSG synthesis had a relatively minor effect on phagocytosis, and the percentage of macrophages with phagocytosed trypanosomes rose only marginally from 2.7 ± 2.3% to 3.9 ± 2.4%. Blocking VSG synthesis in the presence of NMS resulted in a further marginal increase from 5.2 ± 1.9% to 6.7 ± 3.1% (Fig 1B).

However, the most striking effect on phagocytosis was seen when VSG221 synthesis was blocked in trypanosomes which were first opsonised with anti-VSG221 antibodies before the phagocytosis assay was performed. These treatments lead to 33.5 ± 6.7% of the macrophages containing internalised flagella, which was a 3.4-fold increase compared with non-induced cells (**P<0.001). The addition of host complement to the anti-VSG antibodies in the face of a VSG synthesis block did not result in a significant additional increase in phagocytosis (34.8 ± 6.6% vs 33.5 ± 6.7%). This demonstrates that anti-VSG antibodies were the main factor in this facilitated trypanosome uptake after the induction of a VSG synthesis block. Not all macrophages phagocytosed trypanosomes. This variability could indicate that not all macrophages were equally competent for phagocytosis, possibly due to heterogeneity in their degree of activation, as has been found before [32].

**Fig 2:**
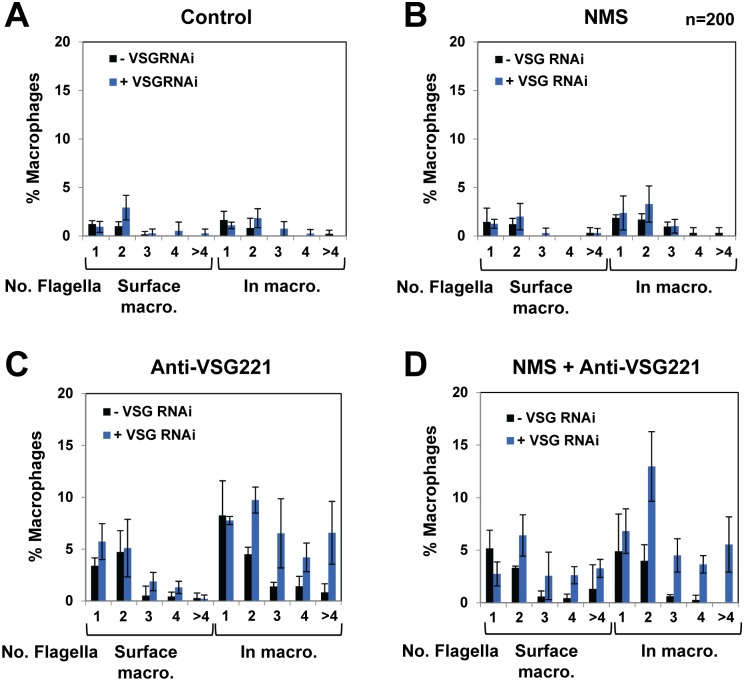
Blocking VSG synthesis in the presence of anti-VSG221 antibodies results in an increase in adherence and phagocytosis of trypanosomes by macrophages. *T*. *brucei* 221VG1.1 was left untreated **(A)**, or opsonised with normal mouse serum (NMS) **(B)**, a polyclonal anti-VSG221 antibody **(C)**, or both NMS and an anti-VSG221 antibody **(D)**, prior to co-culturing with RAW264.7 mouse macrophages. Adherence or phagocytosis of *T*. *brucei* was quantitated by counting the number of flagella either attached to, or inside each macrophage using an antibody against *T*. *brucei* paraflagellar rod protein PFR2 (L8C4). Graphs show mean values expressed as percentage of the total number of macrophages from three independent experiments (n = 200) with the standard deviation indicated with error bars. The number (No.) of flagella either adhering to the surface of the macrophages or internalised within is indicated underneath.

As opsonisation with host proteins appeared to play a significant role in this observed increase in trypanosome recognition and phagocytosis by macrophages, we investigated the extent to which opsonised trypanosomes were actually phagocytosed, as opposed to just adhered to the macrophage surface. When *T*. *brucei* expressing eGFP is phagocytosed, eGFP fluorescence disappears rapidly. We therefore determined the number of trypanosomes internalised in each macrophage using immunofluorescence microscopy detecting the flagella. Trypanosome flagella remain relatively resistant to degradation in the macrophage phagolysosome, even after the ingested trypanosomes have lysed. A detailed analysis in Fig 2 shows that trypanosomes that are pre-incubated in NMS do not adhere to macrophages to a significantly greater extent than untreated trypanosomes regardless of whether *VSG* RNAi is induced (Fig 2A and 2B). Pre-incubation of uninduced *T*. *brucei* with anti-VSG221 antibodies lead to a 4.5-fold increase in RAW264.7 macrophages showing adhered trypanosomes, and a 3.4-fold increase in macrophages with internalised flagella (Fig 2C).

However, the most striking effect was seen when VSG221 synthesis was blocked in trypanosomes which had also been pre-incubated with anti-VSG221 antibodies before co-culturing with macrophages (Fig 2C). Here, the majority of the macrophages contained more than 1 flagellum, and between 5–10% of the macrophages each contained either 1, 2, 3, 4 or more than 4 flagella. Some macrophages efficiently phagocytosed multiple (up to more than four) trypanosomes in the presence of anti-VSG221 antibodies and a VSG synthesis block, with the additional inclusion of NMS resulting in some increased trypanosome adherence and phagocytosis (Fig 2D). Anti-VSG221 antibodies were therefore the most important opsonin facilitating efficient trypanosome uptake in our phagocytosis experiments.

### Clearance of anti-VSG221 antibodies is impaired after the induction of a block in VSG221 synthesis

**Fig 3:**
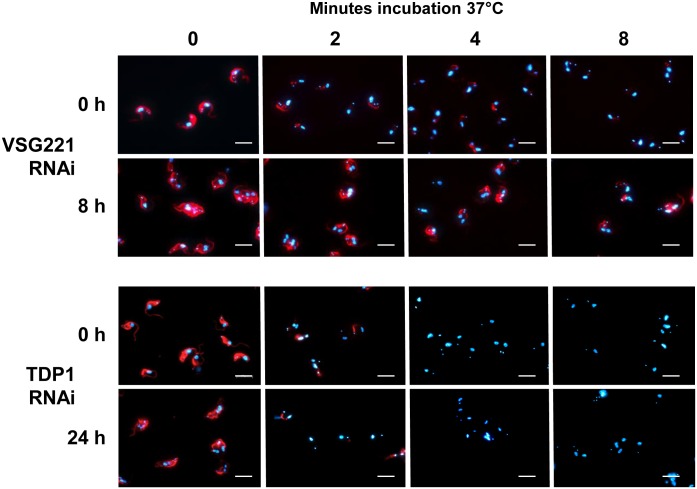
Blocking VSG synthesis results in reduced clearance of surface-bound anti-VSG221 antibodies. Immunofluorescence microscopy analysis of *T*. *brucei* 221VB1.2 where VSG221 synthesis was blocked by the induction of *VSG221* RNAi for 0 or 8 hours (h). Cells were next coated with an anti-VSG221 antibody at 4°C (to stop endocytosis), and subsequently transferred to 37°C for the time indicated in minutes to reinitiate endocytosis. Cells were fixed, and the anti-VSG221 antibody was visualised using an anti-rabbit Alexa 488-conjugated secondary antibody. DNA is stained with DAPI (blue). Scale bar = 10 μm. As a lethality control, a comparable experiment was performed after the induction of RNAi against the essential TDP1 chromatin protein.

Bloodstream form *T*. *brucei* clears anti-VSG antibodies from its surface via unusually high rates of endocytosis, which can be blocked by decreasing the temperature [20, 24]. At 4°C molecules can enter the trypanosome flagellar pocket, but further entry into the endocytic system is halted [24]. To investigate VSG antibody clearance in our stalled cells, we cooled trypanosomes to 4°C to block endocytosis, before allowing binding of anti-VSG221 antibody to the cell surface [24]. The opsonised cells were subsequently transferred to 37°C to reinitiate endocytosis for varying lengths of time. Clearance of surface anti-VSG221 antibodies was visualised using immunofluorescence microscopy. Bloodstream form *T*. *brucei* where *VSG221* RNAi was not induced removed anti-VSG221 antibodies from the cell surface within 8 minutes of reactivation of endocytosis (Fig 3). However, after VSG221 synthesis had been blocked for 8 hours, the rate of surface antibody clearance was greatly reduced. To confirm that we were not looking at a nonspecific consequence of the growth arrest, we also monitored clearance of anti-VSG221 antibodies after the RNAi mediated knock-down of the essential protein TDP1 [37]. TDP is an architectural chromatin protein which has no direct role in VSG trafficking or cellular architecture. The induction of *TDP1* RNAi (although arresting cell growth) did not result in impaired clearance of anti-VSG221 antibodies (Fig 3).

**Fig 4:**
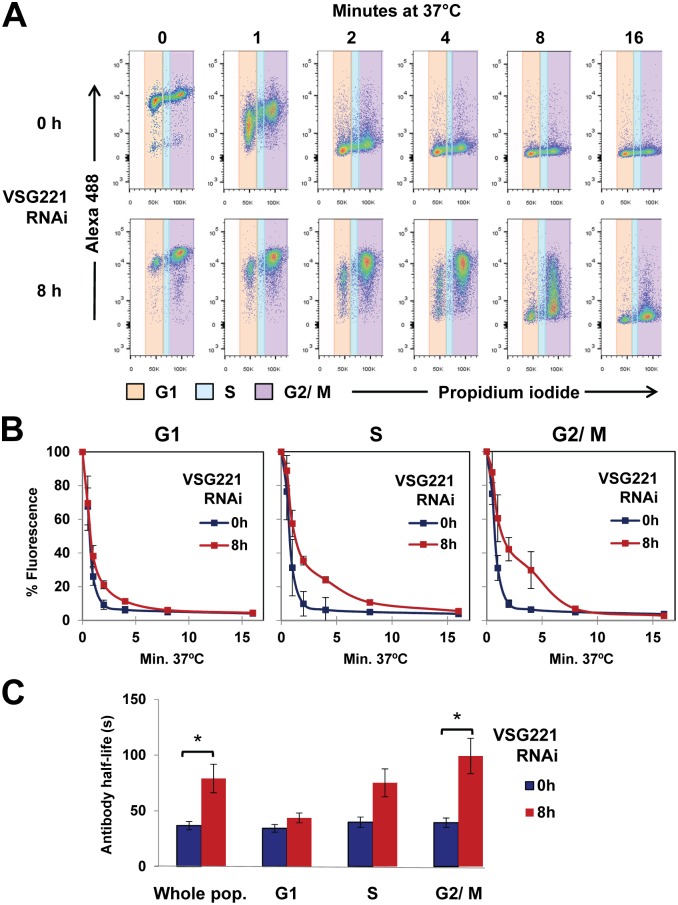
Induction of a VSG synthesis block leads to a decrease in clearance of surface-bound anti-VSG221 antibodies. **(A)** Surface clearance of anti-VSG221 antibodies was analysed by flow cytometry in *T*. *brucei* 221VB1.2 where *VSG221* RNAi was induced for 0 or 8 hours (h). The cells were subsequently transferred to 4°C to stop endocytosis, and then coated with anti-VSG221 antibodies. Cells were next incubated at 37° to reinitiate endocytosis for the time indicated in minutes. The reaction was subsequently stopped, and the cells were fixed and stained with an AlexaFluor 488 coupled secondary antibody and propidium iodide (to visualise DNA content). The amount of surface anti-VSG221 antibody was determined at the G1 (orange), S (blue) or G2/ M (purple) cell cycle stages. **(B)** Quantitation of the reduced clearance of anti-VSG221 antibody after blocking VSG221 synthesis. Surface anti-VSG221 antibody was detected using an Alexa 488-conjugated secondary antibody, and total fluorescence was quantitated by flow cytometry. Mean fluorescence intensity values are expressed as a percentage of the value at 0 minutes. Results shown are the mean of three independent biological replicates with the standard deviation indicated with error bars. **(C)** Quantitation of the half-life of anti-VSG221 antibodies after blocking VSG221 synthesis. Results shown are the mean of three independent biological replicates with the standard deviation indicated with error bars. After fitting each data set to the non-linear regression model, statistical analysis was performed using the Student’s t-test *P<0.05.

Within eight hours induction of *VSG221* RNAi *in vitro*, more than 65% of the population is stalled precytokinesis as 2N2K cells containing two nuclei (N) and two kinetoplasts (K), which do not reinitiate further rounds of DNA synthesis. We therefore investigated if the observed reduced clearance of anti-VSG221 antibodies was a consequence of the accumulation of 2N2K cells. Possibly the 2N2K cell-cycle stage naturally shows reduced rates of recycling. We therefore quantitated the clearance of anti-VSG221 antibodies at different cell cycle stages using flow cytometry (Fig 4A). The anti-VSG221 antibodies were visualised using a secondary antibody coupled to Alexa Fluor 488, and trypanosome DNA content was monitored using the propidium iodide stain.

In untreated cells, we did not find that rates of clearance of anti-VSG221 antibody varied significantly at different stages of the cell cycle (Fig 4A and 4B). The half-life of the anti-VSG antibody in the whole population is 36.7 ± 3.7 seconds (Fig 4C), which is comparable to the value of 35.3 ± 4.6 seconds found by the Engstler laboratory using polyclonal anti-VSG IgG antibodies [24]. When the different cell cycle stages were investigated individually, the half-life of the anti-VSG antibody was comparable between the different cell cycle stages, and ranged from 34.3 ± 3.5 seconds for cells in G1, to 39.8 ± 4.7 seconds for cells in S-phase and 39.3 ± 4.2 seconds for cells at G2/M.

However, in cells that were stalled after blocking VSG synthesis, we found significantly reduced clearance of anti-VSG antibodies at all stages of the cell cycle, with an average for the whole population of 79.0 ± 12.8 seconds) (*P<0.05) (Fig 4). If the different cell cycle stages were analysed individually, the half-life increased from 43.6 ± 4.4 seconds for cells in G1 to 75.1 ± 12.5 seconds for cells in S phase, and 99.2 ± 15.9 seconds for cells in G2/M. In the G2/M cell cycle stage anti-VSG antibody clearance was particularly decreased compared with uninduced cells at this cell cycle stage (*P<0.05). Again, to ensure that we were monitoring a specific consequence of blocking VSG221 synthesis rather than lethality, we also determined the rate of internalisation of anti-VSG221 antibodies after knocking down the highly essential chromatin protein TDP1 [37]. TDP1 knock-down results in rapid growth arrest, although not the highly specific precytokinesis cell cycle arrest observed after the induction of *VSG* RNAi, which is to the best of our knowledge unique [13, 37]. Despite the induction of a rapid growth arrest after TDP1 knock-down, there was no significant change in the rate of clearance of anti-VSG221 antibodies (41.1 ± 4.0 seconds) (S3 Fig).

A phenomenon that we observed in these experiments, was that cells where *VSG221* RNAi had been induced, appeared to have increased binding of anti-VSG221 antibody compared with uninduced cells. We therefore quantitated the total amount of VSG221 on these stalled cells using LiCor imaging. We found that *T*. *brucei* where *VSG221* RNAi had been induced for eight hours had a reduction in total VSG to 64 ± 12% normal levels (*P<0.05) (S4A and S4B Fig). We also quantitated surface VSG using biotinylation. These biotinylation experiments estimated that *T*. *brucei* had a reduction in surface VSG221 to 67 ± 22% normal levels after the induction of *VSG221* RNAi for eight hours (*P<0.05) (S4C Fig). Equivalent values were obtained if the different cell cycle stages were analysed separately. However, if anti-VSG221 antibodies were quantitated, approximately twice as many bound to the surface of cells where *VSG* RNAi had been induced (*P<0.05) (S4D Fig). This was particularly apparent in the G2/ M cell cycle stage (213 ± 41%). As we had established that blocking VSG synthesis resulted in moderate reduction in VSG on the cell surface, this observed increase in antibody binding is presumably a consequence of reduced VSG coat density exposing previously inaccessible VSG epitopes to recognition by the polyclonal anti-VSG antibodies.

**Fig 5:**
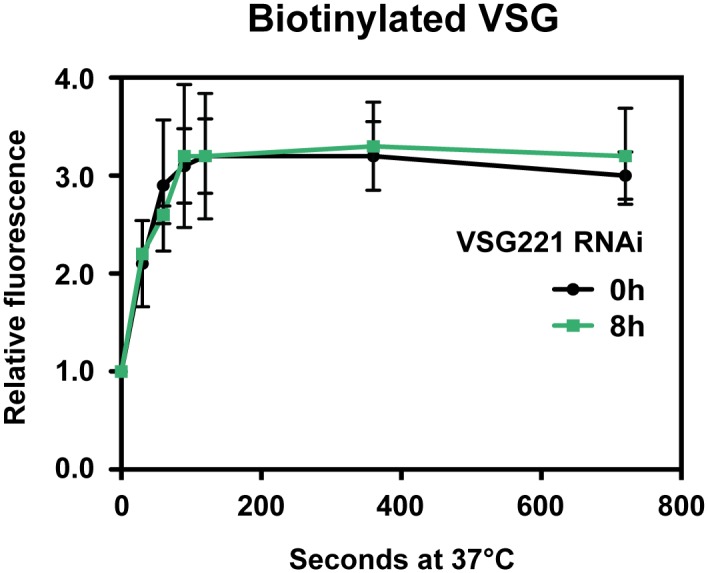
The rate of internalisation of biotinylated VSG is unaffected by the induction of a block in VSG synthesis. VSG synthesis was arrested in *T*. *brucei* 221VB1.2 cells by the induction of *VSG221* RNAi for 8 hours (h). Endocytosis was stopped by cooling the cells to 4°C. Flow cytometry analysis of internalisation of biotinylated VSG. Cells were labelled with biotin, and transferred to 37°C for 0, 30, 60, 90, 180, 360 or 720 seconds to activate endocytosis. Remaining surface biotin was removed by the addition of ice cold stripping buffer. Cells were next fixed, permeabilised and incubated with Alexa Fluor 488 conjugated streptavidin. Mean fluorescence intensity values were normalised against that of time point 0 seconds. Error bars show the standard deviation of three independent biological replicates.

As we observed reduced clearance of anti-VSG antibodies after induction of a VSG synthesis block, we investigated if this effect was due to a reduction in the rate of internalisation of total surface VSG rather than specifically antibody bound VSG. Cells were cooled to 4°C to stop endocytosis, before surface VSG was biotinylated [20]. Cells were subsequently transferred to 37°C to reinitiate endocytosis for varying lengths of time. Endocytosis was then stopped, external biotin was stripped off, and fixed cells were permeabilised and incubated with streptavidin-Alexa Fluor 488 to quantitate the amount of internalised biotinylated VSG by flow cytometry. However, even after blocking VSG synthesis for 8 hours, the amount of surface VSG that was internalised was not significantly decreased (half-life of 23.7 ± 6.1 vs 28.4 ± 7.5 seconds) (Fig 5). This demonstrates that in cells arrested by the induction of *VSG221* RNAi, there is specifically a reduction in the rate of internalisation of VSG-antibody complexes, rather than a general decrease in rates of internalisation of all surface VSG.

Although anti-VSG221 antibodies were clearly removed from the cell surface at a reduced rate after the induction of *VSG221* RNAi, it was not evident whether this impairment was a consequence of a restriction preventing anti-VSG antibody complexes from entering the flagellar pocket, or complications further downstream in the endocytic pathway. We therefore monitored if the VSG-antibody complexes were indeed internalised using immunofluorescence microscopy. Similar to previous experiments, cells were first cooled to 4°C to block endocytosis, before transferral to 37°C for varying lengths of time. Reactivation of endocytosis at 37°C results in VSG-antibody complexes progressing first through the early, and then subsequently the late endocytic system. In the lysosomal compartment anti-VSG antibodies are stripped off and degraded [23, 38]. In order to prevent degradation of the internalised VSG-antibody complexes in the lysosome and thereby facilitate their detection, FMK-024 inhibitor was included at an end concentration of 20 μM. FMK-024 is a lysosomal thiol protease inhibitor which blocks the cathepsin protease TbCat L, allowing the detection of accumulated anti-VSG antibodies in the *T*. *brucei* lysosome [39, 40].

**Fig 6:**
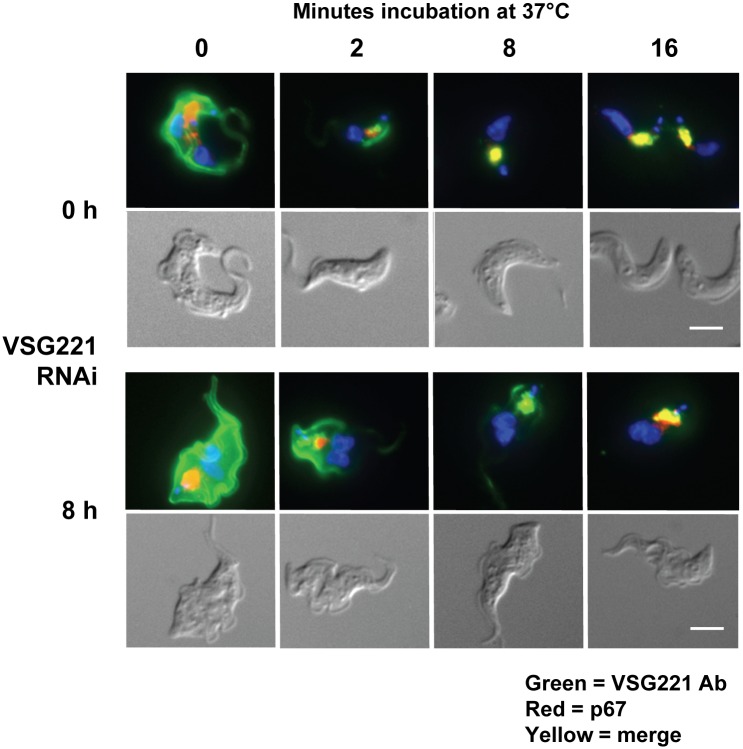
Blocking VSG synthesis results in impairment of internalisation of anti-VSG antibodies. Reduced rates of internalisation and lysosomal degradation of anti-VSG221 antibody after induction of a VSG221 synthesis block. VSG221 synthesis was blocked in *T*. *brucei* 221VB1.2 cells by the induction of *VSG221* RNAi for 8 hours (h). Cells were coated with an anti-VSG221 antibody at 4°C to stop endocytosis, and then transferred to 37°C to activate endocytosis for the time shown in minutes. The lysosomal thiol protease inhibitor FMK-024 was used to inhibit degradation of anti-VSG antibody in the lysosomal compartment, and facilitate its visualisation. Cells were then fixed and stained with an Alexa Fluor 488 coupled secondary antibody. The lysosomal compartment was visualised with an antibody against the p67 lysosomal protein stained with an Alexa Fluor 594 coupled secondary antibody. The merge of the two signals is shown in yellow. Below are shown panels of the trypanosomes visualised using differential interference contrast. Scale bar: 5 μm.

As expected, after transferring cells to 37°C anti-VSG antibodies (indicated in green) were rapidly removed from the cell surface within 2 minutes (Fig 6). The *T*. *brucei* lysosomal compartment was visualised with the lysosomal marker p67 (red) [41, 42]. In untreated cells, anti-VSG antibodies accumulated in the lysosome within 8 minutes of reactivation of endocytosis (co-localisation with p67 indicated in yellow). After blocking VSG synthesis, anti-VSG antibodies were still internalised but at a greatly reduced rate, and it took more than 16 minutes before significant accumulation of antibodies in the lysosome was observed.

**Fig 7:**
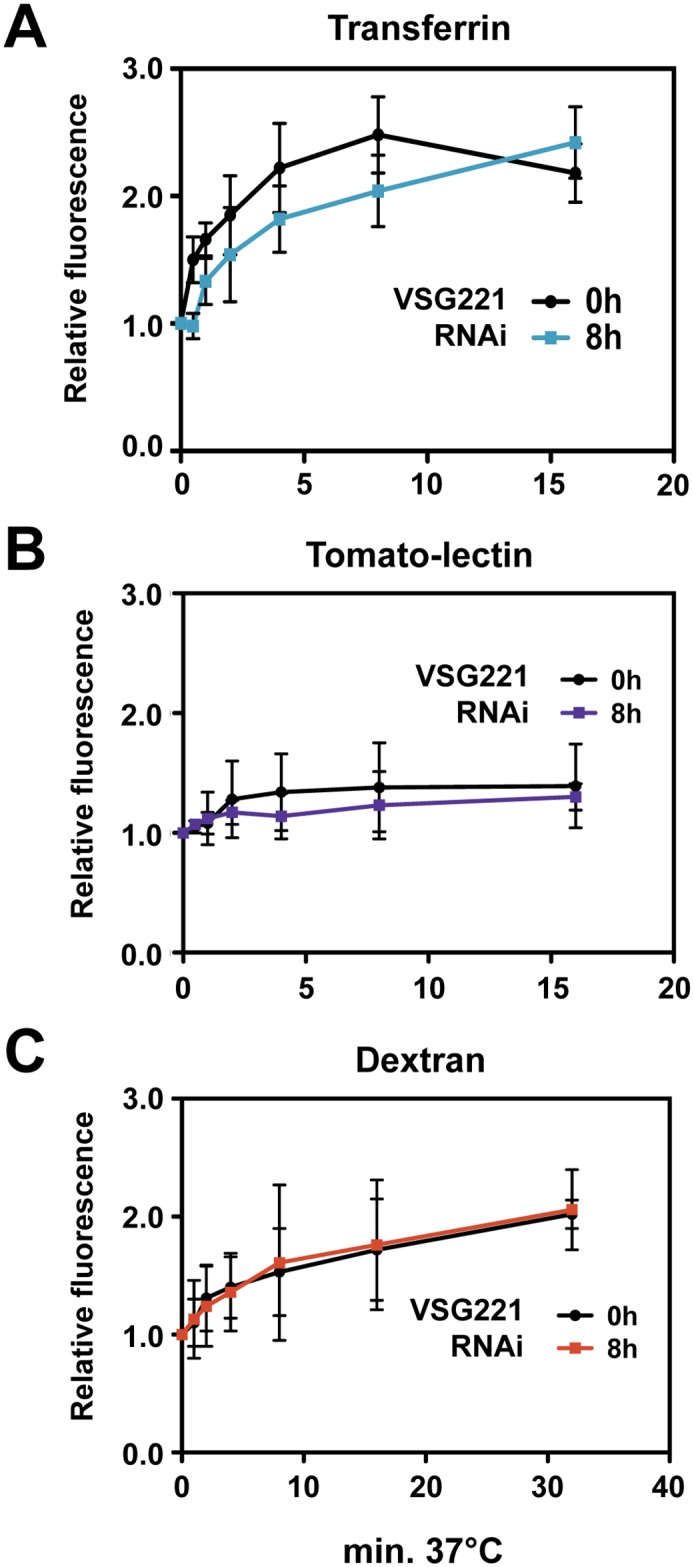
Blocking VSG synthesis does not result in significant reduction in rates of endocytosis of transferrin, tomato lectin or dextran. **(A)** Internalisation of transferrin after the induction of a VSG synthesis block. Cells were incubated with Alexa Fluor 488-conjugated transferrin at 4°C and then transferred to 37°C to reinitiate endocytosis for the time indicated in minutes. Cells were subsequently fixed and analysed by flow cytometry. Mean fluorescence intensity values are normalised to that at 0 minutes. The standard deviation from three independent experiments is indicated with error bars. **(B)** Uptake of tomato-lectin after the induction of a VSG synthesis block. The experiment was performed and analysed by flow cytometry as in **(A)** only cells were incubated with tomato lectin coupled to Dylight 488 at 4°C before reactivation of endocytosis at 37°C for the time indicated in minutes. **(C)** Flow cytometry analysis of dextran uptake after blocking VSG synthesis. Cells were incubated with Alexa Fluor 488 conjugated dextran at 4°C and transferred to 37°C for the time indicated in minutes. Mean fluorescence intensity values were normalised against that of the time point 0 minutes. The standard deviation of three independent biological replicates is indicated with error bars.

Endocytosis in bloodstream form *T*. *brucei* is restricted to the flagellar pocket, and is involved in the removal of surface bound host antibodies, nutrient uptake and the maintenance of homeostasis of the cell surface proteome. In order to determine if the early endocytic pathway was affected in our stalled cells, we investigated rates of receptor mediated endocytosis by following the uptake of transferrin and tomato lectin, and fluid phase endocytosis by monitoring uptake of dextran. Using flow cytometry to monitor uptake of Alexa Fluor 488 conjugated human transferrin, we found that the half-life of transferrin increased from 1.1 ± 0.3 minutes to 3.3 ± 1.1 minutes, however this result was not statistically significant (Fig 7A). Uptake of other endocytic makers was also not significantly affected by the induction of *VSG* RNAi. Receptor-mediated endocytosis was monitored with tomato-lectin coupled to Alexa Fluor 488, which showed an increase in half-life (3.5 ± 4.2 minutes versus 1.5 ± 1.3 minutes), but this was not statistically significant (Fig 7B). Similarly, fluid phase endocytosis, as monitored by uptake of Dextran-Alexa Fluor 488 was also not significantly changed in the stalled cells (7.9 ± 6.3 minutes in cells where *VSG* RNAi had been induced compared with a half-life of 8.3 ± 5.0 minutes in untreated cells) (Fig 7C). These results therefore do not provide evidence for a clear impairment in the early endocytic pathway in cells stalled after the induction of a VSG synthesis block.

### Blocking VSG synthesis results in increased directional cell motility and reduced tumbling

Motility is essential in bloodstream form *T*. *brucei* and plays a vital role in cell cycle progression, cytokinesis and virulence [43, 44]. Forward swimming motility is also thought to facilitate the removal of anti-VSG antibodies from the surface of the bloodstream form trypanosome [24]. In this model, antibodies bound to the VSG function as molecular sails. Hydrodynamic forces generated by forward swimming of the parasite contribute to the preferential backward movement of VSG-antibody complexes into the flagellar pocket [24]. As motility is thought to be key for efficient surface antibody clearance, we examined how this is affected after VSG synthesis is blocked.

**Fig 8:**
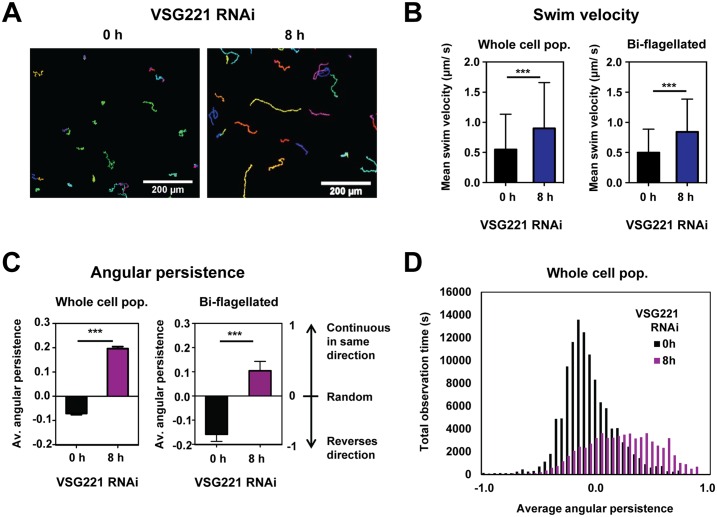
Motility analyses show an increase in swim velocity in cells where VSG synthesis has been inhibited. **(A)** *T*. *brucei* swims more persistently in the same direction after VSG synthesis is blocked. Particle motion tracks of cells where *VSG221* RNAi was induced for 0 or 8 hours (h). Each track represents the distance travelled by an individual cell over the course of measurement. One image was captured every 200 ms (5 Hz) for 1.7 minutes. Scale bar: 200 μm. **(B)** There is a significant increase in swim velocity (or directional swimming) after the induction of a VSG synthesis block for 8 hours. Velocity is shown in μm per second (s). The whole population (2500 cells) is compared with 50 bi-flagellated cells from the induced or uninduced population. Statistical significance was determined with Student’s t-test (***P<0.0001). **(C)** Increase in angular persistence in cells where VSG synthesis has been blocked. The average (av.) angular persistence is shown in either the whole cell population or bi-flagellated cells. Angular persistence is denoted on an arbitrary continuous scale of -1 to 1 where a value of 1 indicates continuous swimming in the same direction as observed after a 2 second period, a value of 0 represents cells moving in a random direction after this time period, and a value of -1 represents a trypanosome observed swimming in the reverse direction after 2 seconds. 1500 cells from the whole population (pop.) and 50 bi-flagellated cells are analysed. The observed change in motility after the induction of a VSG synthesis block was highly significant (***P<0.0001). **(D)** Histogram distribution showing an increase in persistent directional swimming in cells where VSG synthesis was blocked. The data is from 1500 cells from the whole population (either untreated or where *VSG221* RNAi has been induced for 8 hours) which are also analysed in panel **(C)**.

We measured the motility of our cells by performing time-lapse live cell videomicroscopy, whereby cells were observed by bright-field light microscopy and images were captured every 200 milliseconds for 1.7 minutes (512 frames). Particles were identified, and tracks were built for each cell (Fig 8A). VSG synthesis was blocked by the induction of *VSG* RNAi for 8 hours. Analysis of the whole population did not show a significant change in average trypanosome swim speed after the induction of *VSG* RNAi (1.6 ± 0.014 μm/ second vs 1.7 ± 0.019 μm/ second) (S5 Fig). Arresting VSG synthesis resulted in a population of stalled trypanosomes that were predominantly (>65%) composed of cells that had arrested precytokinesis with two full length flagella. We therefore next monitored the speed of bi-flagellated cells in the untreated population, or after VSG221 synthesis had been blocked for 8 hours. Here too, there was no significant change in swim speed after the induction of a VSG synthesis block (1.45 μm/ sec vs 1.58 μm/ sec) (S5 Fig).

However, in the population of cells where VSG synthesis had been blocked, there was a clear difference in swimming behaviour as illustrated by the particle motion track plots (Fig 8A). *T*. *brucei* motility changes according to the viscosity of its environment [24, 45]. Although tissue culture medium is not as viscous as blood, we still used this low viscosity medium in our motility experiments in order to replicate the conditions used in the trypanosome macrophage co-cultivation experiments. In the uninduced population, tumbling cells constituted the majority of the population, as has been observed earlier for bloodstream form *T*. *brucei* cultured under standard tissue culture conditions [24]. However, after blocking VSG synthesis for 8 hours, the arrested trypanosomes clearly swam in a more persistently directional fashion.

We therefore measured average trypanosome velocity, which is defined as speed in a given direction, or total displacement over track length (and therefore including a directional component). In contrast, average speed is total distance travelled over track length without considering directionality. After blocking VSG synthesis for eight hours, the average trypanosome velocity significantly increased from 0.55 ± 0.01 μm/s to 0.90 ± 0.02 μm/s (***P = <0.0001) reflecting the increased directionality of movement (Fig 8B). Again, we investigated if this was due to the accumulation of bi-flagellated cells in our arrested population, but found a similar increase in velocity (or directional swimming) after the induction of a VSG synthesis arrest in these bi-flagellated cells from 0.50 ± 0.06 μm/s to 0.84 ± 0.08 μm/s (***P = <0.0001).

These results were confirmed by our measurements of average angular persistence. Angular persistence is a mathematical description for how the direction of motion of the cell changes over time [46]. A value ranging from -1 to 1 was assigned to each particle track corresponding to a swimming trypanosome. A value of 1 indicates directional swimming, whereby cells observed after a 2 second period swim in the same direction as in the previous 2 second period. A value of 0 represents random movement, whereby the movement of the cell observed after a 2 second period is completely random in direction compared with the previous 2 second period. A value of -1 indicates a reversal in swimming direction, whereby the cell observed after a 2 second period has moved in the reverse direction.

As seen in the particle tracks, bloodstream form *T*. *brucei* primarily tumbles under standard *in vitro* culturing conditions (with average angular persistence values of -0.07 ± 0.006) (Fig 8C). However, after VSG synthesis is blocked, the average angular persistence increased to 0.20 ± 0.008 units, reflecting the striking shift in the predominant mode of swimming from random to directional (***P<0.0001). Again, this change in motility after the induction of a VSG synthesis arrest was observed in both the whole population as well as in bi-flagellated cells. In bi-flagellated cells the angular persistence increased after the induction of *VSG* RNAi (-0.18 ± 0.03 units to 0.10 ± 0.04) which is significantly higher than in bi-flagellated cells in the untreated population (***P<0.0001).

At the population level, the heterogeneity of motility phenotypes was greater in cells where VSG synthesis was blocked compared with the non-induced population, possibly due to variance in penetrance of the *VSG RNAi* phenotype between cells (Fig 8D). However, in general there was a clear increase in directional swimming in cells where VSG synthesis had been blocked. We have shown earlier that blocking VSG synthesis results in stalled cells which are shorter and broader than normal, although the cell volume remains unchanged [13]. We postulated that this changed morphology could be a consequence of the cell attempting to minimise its surface area-to-volume ratio (i.e. become more spherical) in the face of restricted VSG. One would expect that cells with a restricted VSG coat would also be more rigid than normal. This could be expected to reduce their ability to flex, which could explain the reduced tumbling movement that we observe.

These motility changes were striking, and could possibly explain the observed reduction in clearance of anti-VSG antibodies. It is possible that cell surface flexing on the tumbling trypanosome generates localised hydrodynamic forces facilitating the movement of VSG-antibody complexes toward the flagellar pocket. These forces could be as significant for immune complex removal as those operating on a directionally swimming trypanosome. Therefore the motility changes that we observe in cells where VSG synthesis has been blocked, possibly play a direct role in the reduced selective clearance of anti-VSG complexes from the cell surface.

In summary, these results highlight the importance of efficient removal of anti-VSG antibodies from the trypanosome cell surface to allow *T*. *brucei* evasion from phagocytosis of host macrophages, and identify the rapid removal of host opsonins as being a key virulence factor in a trypanosome infection.

## Discussion

Here, we investigate the role of the VSG coat and its recycling in the phagocytosis of *T*. *brucei* by macrophages. We find that blocking VSG synthesis results in a significant increase in macrophage phagocytosis of trypanosomes, but only in the presence of anti-VSG antibodies. Blocking VSG synthesis for 8 hours resulted in reduced rates of clearance of anti-VSG antibodies. This was particularly pronounced in G2/ M stage cells, where the half-life of the anti-VSG antibody increased about two and a half fold from 39.3 ± 4.2 seconds to 99 ± 15.9 seconds. There was specifically a perturbation in the removal of VSG-antibody complexes, as rates of internalisation of total biotinylated surface VSG were not significantly changed after blocking VSG synthesis. There was no evidence for a restriction operating around the flagellar pocket of the stalled cells, as VSG antibody still reached the lysosomal compartment after a VSG synthesis block, although at slower rates than normal. Rates of endocytosis of various endocytic markers including tomato lectin, dextran or transferrin were not significantly reduced in the stalled cells. Motility was altered in the arrested cells, resulting in cells which swam persistently rather than tumbling. We postulate that these motility changes play a direct role in the reduced clearance of anti-VSG antibodies that we observe. These experiments shed light on *T*. *brucei* evasion of macrophages. In addition, they highlight the essentiality of high rates of VSG synthesis and internalisation coupled with VSG quality control in removing host cell opsonins, thereby enabling trypanosome escape from host phagocytes.

The experiments where trypanosomes were co-cultivated with macrophages demonstrated how effectively wild-type bloodstream form *T*. *brucei* normally evades ingestion by phagocytes. After macrophages had been co-cultivated with *T*. *brucei* for 60 minutes, only a small percentage (2.7 ± 2.3%) had ingested trypanosomes. Phagocytosis was increased in the presence of host opsonins. The addition of anti-VSG antibody produced the most striking increase, with the addition of mouse complement having a relatively minor effect. However, the highest (and statistically significant) levels of phagocytosis were observed after pre-treatment of the trypanosomes with both anti-VSG antibodies as well as complement (with 16.4 ± 6.0% of the macrophages showing internalised flagella). These results therefore highlight the importance of host opsonins, and specifically the classical pathway of complement activation in the recognition and subsequent phagocytosis of trypanosomes by professional phagocytes including macrophages, [26, 31, 47].

Our experimental approach investigates trypanosome removal of anti-VSG221 antibodies derived from hyperimmune polyclonal rabbit serum, which can be expected to be highly enriched for the IgG isotype. In contrast, the IgM isotype would be likely to be more abundant early during a trypanosome infection, although its relative importance for trypanosome clearance has been disputed [48]. Both antibody isotypes can be efficiently removed by *T*. *brucei* from the cell surface [23, 24], and we do not think that these differences significantly impact on our findings.

Macrophages have a variety of surface receptors for these host molecules, including different types of Fc receptors which bind the Fc regions of IgM or IgG antibodies coating microorganisms [26, 49]. Alternatively, macrophage binding can operate through complement proteins deposited through either the alternative or the classical pathway of complement activation. In the alternative pathway, complement C3 is cleaved, and the opsonin C3b plus its degradation products including iC3b are deposited directly on the microbial surface [35]. Monocytes and macrophages have different surface receptors for these host proteins. These include CR1, CR3 and CR4 which bind the C3b and iC3b coated microorganisms, thereby facilitating recognition and phagocytosis [34]. However, in the classical pathway, the complement protein C1q binds the Fc region of IgG or IgM antibodies coating microorganisms, which can then be recognised by macrophage receptors including CR1 [34, 50]. The synergistic effect of adding both anti-VSG antibodies and normal mouse serum on trypanosome phagocytosis that we observed, is presumably a consequence of improved macrophage recognition of parasites opsonised with antibodies as well as different types of complement proteins (including C3b, iC3b and C1q) indicating the importance of the classical pathway. These experiments emphasise the vital importance for *T*. *brucei* of rapidly removing host opsonins from its surface, before these can be recognised by macrophage cell surface receptors.

Blocking VSG synthesis for 8 hours did not result in a statistically significant increase in macrophage phagocytosis of *T*. *brucei*, despite this resulting in the preponderance of the *T*. *brucei* population (>65%) arresting precytokinesis as stalled bi-flagellated cells. This was not significantly affected by the addition of normal mouse serum. However, in the presence of anti-VSG antibodies, there was a striking increase in macrophage phagocytosis of trypanosomes, with up to 34% of the macrophages containing internalised flagella. Activated macrophages were able to internalise multiple trypanosomes, with many phagocytes ingesting more than four parasites. Even under optimal conditions, not all macrophages phagocytosed trypanosomes. This was presumably a consequence of heterogeneity in macrophage activation state after treatment with LPS and interferon γ, as appears to have been observed earlier in the mouse RAW264.7 cell line used in these studies [32].

We show that the increased phagocytosis of trypanosomes in the presence of anti-VSG antibodies after the induction of a VSG synthesis block, appears to be a direct consequence of a reduction in rates of antibody clearance from the trypanosome cell surface. After the induction of *VSG221* RNAi, VSG antibody half-life on the cell surface increased significantly from 36.7 ± 3.7 seconds to 79.0 ± 12.8 seconds. This was most striking in G2/ M cells where the surface anti-VSG antibody half-life increased about two and a half fold from 39.3 ± 4.2 to 99.2 ± 15.9 seconds. Surprisingly, this reduced clearance of anti-VSG antibody in the arrested cells did not appear to be a consequence of reduced rates of internalisation of bulk surface VSG. Similarly, there did not appear to be a restriction operating on flagellar pocket access. After blocking VSG synthesis, surface anti-VSG antibody could still enter the pocket and progress through the endocytic system to the lysosome, although at slower rates than normal.

Endocytosis at the flagellar pocket in bloodstream form *T*. *brucei* involves clathrin coated pits which bud off the cellular membrane and shed their clathrin lattice, forming early endosomes [51–53]. Although we found that blocking VSG synthesis resulted in decreased clearance of anti-VSG antibodies from the cell surface, we did not find significant disruption in rates of endocytosis of either bulk VSG or various markers for either receptor mediated or fluid phase endocytosis. This would indicate that the reduced clearance of surface antibodies that we observed was not due to a clear endocytic defect.

However, the induction of a VSG synthesis block did result in significant changes in the motility of the stalled cells. Trypanosome swim speeds are thought to be instrumental for the efficient removal of anti-VSG antibodies from the cell surface [24], which is favoured at swim speeds much higher than those observed under standard *in vitro* culturing conditions which are lower viscosity than blood [24, 54]. The swim speed of bloodstream form *T*. *brucei* 427 grown at 37°C under standard tissue culture conditions varies between 1.5 to 5.7 μm/ sec [54, 55]. Although our observed swim speed is low (1.5 μm/ sec), we did not repeat these experiments in high viscosity media, as we were interested in *T*. *brucei* motility under the conditions used for the macrophage co-cultivation experiments.

Populations of bloodstream form *T*. *brucei* typically contain cells with a variety of different swimming behaviours varying from persistent directional swimming to tumbling [56, 57]. Cell microenvironment affects this motility, whereby trypanosomes cultured in the low viscosities of tissue culture media predominantly tumble [54]. In *T*. *brucei* swimming becomes faster and more directional as the viscosity of the culture medium is increased up to values found in blood [45, 54]. In addition to the effect of the microenvironment, cellular features have been argued to affect trypanosome motility as straighter rigid cells would be expected to swim more directionally [57].

Our bloodstream form *T*. *brucei* showed primarily tumbling behaviour *in vitro* as would be expected under low viscosity conditions, but the induction of a VSG synthesis block resulted in a significant increase in the number of cells which swam directionally. There was a significant increase in velocity (or directional swimming) of these stalled cells, although the mean swim speed did not significantly increase either in the whole population or in bi-flagellated cells which comprise the majority of the population (>65%). Cells stalled after the induction of a VSG synthesis block arrest at a precise precytokinesis stage, although these stalled cells differ from wild type precytokinesis cells. The arrested cells are shorter and broader than normal [13], although the total cell volume is unchanged compared with normal precytokinesis cells. This change in morphology in the absence of sufficient VSG could be a consequence of a restriction operating on the VSG surface coat, resulting in increased cellular rigidity. As increased cellular rigidity (as well as an increase in cell width) would be expected to result in increased directional swimming [57], this could explain why our stalled bi-flagellated cells swim more directionally than wild type bi-flagellated cells.

Selective clearance of VSG-antibody complexes is thought to be facilitated by hydrodynamic forces operating on the trypanosome cell surface generated by directional swimming [24]. However, even in tumbling cells there is posterior accumulation of anti-VSG immune complexes at the flagellar pocket. Our uninduced cells predominantly tumble, yet clear anti-VSG antibodies, showing that directional swimming is not essential for all antibody clearance. The induction of a VSG synthesis block results in directionally swimming cells with reduced antibody clearance. It is therefore possible that the flexing activity on the cell surface of a tumbling trypanosome generates localised hydrodynamic forces which directly facilitate the selective movement of VSG-antibody complexes over the cell surface. These forces could be as important in removing surface VSG-antibody complexes as the forces generated through directional swimming through the “wind in the sail” model [24]. Our observed changes in motility possibly directly explain why there is decreased removal of VSG-antibody complexes after the induction of VSG RNAi. This would argue that the exact characteristics of *T*. *brucei* motility in the bloodstream could play an important role in removing immune complexes, and therefore in parasite virulence.

Our results therefore shed light on several issues. First of all, they highlight the importance of continued VSG synthesis for the bloodstream form parasite in immune evasion. Blocking VSG synthesis results in viable stalled cells with intact VSG coats which persist for days *in vitro*. This lead to the proposal that this cell cycle arrest could be a parasite safety mechanism preventing a trypanosome from dividing in the absence of enough VSG to coat the two daughter cells. This could potentially have a protective function *in vivo*. However, the protective nature of this cell-cycle checkpoint would only operate in the absence of significant anti-VSG antibody titres. The experiments that we show here, argue that in a host that has been infected long enough to develop a significant antibody response, these stalled cells would be preferentially removed by phagocytes as a consequence of reduced clearance of anti-VSG antibodies.

Secondly, our results directly demonstrate the essentiality of rapid removal of host opsonins from the trypanosome cell surface for effective escape from host macrophages. To the best of our knowledge, these experiments are the first to show that an experimental perturbation leading to reduced clearance of anti-VSG antibodies directly results in increased trypanosome phagocytosis by macrophages. Lastly, we hypothesise that the motility changes observed after induction of a VSG synthesis block play a direct role in VSG-immune complex removal. These experimental data therefore highlight the importance of high rates of VSG recycling in the removal of host cell opsonins from the trypanosome cell surface, thereby allowing the efficient evasion of professional phagocytes including macrophages during a chronic infection. We therefore show that the cellular processes of high rates of VSG internalisation coupled with VSG quality control are key virulence factors enabling *T*. *brucei* to be a highly successful extracellular pathogen.

## Materials and Methods

### Trypanosome strains and culturing

Bloodstream form (BF) *Trypanosoma brucei brucei* 427 was cultured in HMI-9 medium supplemented with 15% heat inactivated fetal calf serum (FCS) [58]. The BF *T*. *brucei* 221VG1.1 cell line used in the phagocytosis assays is described in [13]. This cell line is a derivative of *T*. *brucei* 90–13 [59]. It expresses VSG221, and has a 221GP1 construct containing the *eGFP* and puromycin resistance genes inserted behind the promoter of the active *VSG221* expression site [60]. Selection on puromycin ensures maintenance of a population of cells homogeneous for expression of VSG221. A construct allowing tetracycline inducible expression of *VSG221* RNAi from two opposing T7 promoters is inserted in a mini-chromosome [61].

The similar BF *T*. *brucei* 221VB1.2 cell line is also described in [13], only here a construct containing a blasticidin resistance gene was inserted immediately behind the active *VSG221* expression site promoter, and homogeneous expression for VSG221 was ensured using blasticidin selection. The *T*. *brucei* 221VB1.2 cell line was used for the surface VSG-antibody clearance assays, endocytosis assays and motility measurements. In both cell lines, *VSG221* RNAi was induced for 8 hours using 1 μg/ ml tetracycline. Control experiments were performed with the VSG221 expressing BF *T*. *brucei* 90-13TDPC1 cell line. This is a derivative of BF *T*. *brucei* 90–13 [59] with a fragment from the essential *TDP1* gene inserted between two opposing T7 promoters, allowing the tetracycline inducible expression of *TDP1* RNAi [37].

### Macrophage culture and trypanosome phagocytosis assay

Murine macrophage cell line RAW264.7 (ATCC TIB-71) (kind gift of Prof. Murray Selkirk, Imperial College London) [62] was cultured in DMEM (Dulbecco’s Modified Eagle’s Medium) supplemented with 10% FCS, 1% glutamine and 10,000 U/ ml penicillin-streptomycin. Macrophages were plated onto glass coverslips (22x 22 mm) in 6-well plates (1x 10^6^ per well), and allowed to adhere for two hours at 37°C. In order to trigger classical macrophage activation [31], macrophages were primed with LPS (Lipopolysaccharides *E*. *coli*) at 100 ng/ ml and mouse IFN-γ (interferon gamma) at 20 ng/ ml 24 hours prior to co-cultivation with trypanosomes [32].

For the macrophage phagocytosis experiments, *T*. *brucei* 221VG1.1 (3 x 10^6^) was opsonised with 10% normal mouse serum (NMS) for 30 minutes at 37°C in HMI-9 medium supplemented with 15% FCS. Complement-rich NMS was obtained from CD-1 mice, and is a kind gift of Prof. Paul Morgan (University of Cardiff). CD-1 mice are wild type for complement C5 as well as other components of the terminal complement pathway, and have functional serum complement activity of the classical, lectin and alternative complement pathways at the level of C9 activation (S1 Fig) [36, 63]. Alternatively, trypanosomes were incubated with a 1:5000 dilution of polyclonal rabbit anti-VSG221 antibody (gift of Jay Bangs, SUNY Buffalo) in the presence or absence of NMS for 30 minutes at 37°C. This antibody dilution is not in excess (S2 Fig). The host opsonins (either complement or antibodies) were not removed before incubation with mouse macrophages.

During the macrophage phagocytosis assay, trypanosomes were co-cultured with RAW264.7 macrophages for one hour at 37°C. Culture medium was removed from each well, and the cells were fixed immediately with 2% paraformaldehyde (PFA) for 20 minutes at room temperature. Cells were subsequently washed with PBS buffer, and permeabilised in methanol at -20°C for 30 seconds. *T*. *brucei* flagella were next stained with a mouse monoclonal anti-PFR2 antibody (L8C4) (gift of Keith Gull, University of Oxford) [33], and an anti-mouse Alexa Fluor 594 antibody (Molecular Probes, Life Technologies. The coverslips were removed, mounted onto glass slides and analysed by immunofluorescence microscopy.

### Immunofluorescence microscopy and flow cytometry

Fixed cells were mounted in Vectashield mounting medium containing DAPI (1.5 μg/ ml) (Vector Laboratories). Microscopic analysis was performed using a Zeiss Imager.M1 microscope. Images were obtained using a Zeiss AxioCam Mrm camera and analysed using Zeiss AxioVision4.8 software. ImageJ was used for image analysis [64]. For flow cytometry, fixed cells were permeabilised with 0.1% Triton X-100 for five minutes at room temperature and washed in PBS buffer. Cells were next incubated with RNAse A (1 μg/ ml) and propidium iodide (10 μg/ ml) to stain for DNA, before resuspension in PBS buffer for flow cytometry analysis. Cells were analysed using a BD LSRFortessa cell analyser and analysis was performed using FlowJo software.

### Surface VSG-antibody clearance and biotinylated VSG uptake assays

*T*. *brucei* 221VB1.2 was cooled to 4°C to block endocytosis, and incubated with a 1:5000 dilution of rabbit polyclonal anti-VSG221 antibody for one hour to coat the cells with this antibody. Cells were subsequently transferred to 37°C to reactivate endocytosis for 0, 0.5, 1, 2, 4, 8 or 16 minutes and then diluted in trypanosome dilution buffer (TBD)(5 mM KCl, 80 mM NaCl, 1 mM MgSO_4_, 20 mM Na_2_HPO_4_, 2 mM NaH_2_PO_4_, and 0.36% glucose, pH 7.4). Cells were then fixed in 2% PFA for 20 minutes at room temperature, and analysed by flow cytometry or immunofluorescence microscopy. Clearance of surface VSG-antibody was expressed as the half-life in the decrease in fluorescence from the trypanosome cell surface. Quantitation of surface VSG-antibody clearance was calculated from flow cytometry data (three independent biological replicates) as described in [24].

The protocol used to visualise internalisation of anti-VSG221 antibodies using immunofluorescence microscopy is described in [39]. In order to prevent degradation of anti-VSG221 antibodies in the lysosome, *T*. *brucei* 221VB1.2 cells were incubated with FMK-024, an inhibitor of cathepsin B and L (morpholinourea-phenylalanine-homophenylalanine-fluoromethylketone)(MP Biochemicals) at an end concentration of 20 μM for 1 hour at 37°C [39]. Cells were subsequently cooled to 4°C in 1% BSA/ TDB and coated with rabbit polyclonal anti-VSG221 antibody for 1 hour. Cells were subsequently transferred to 37°C for 0, 8, 16 and 32 minutes. Immediately after incubation, cells were washed in TDB and fixed in 2% paraformaldehyde and permeabilised using 0.1% Triton X-100 at room temperature. Cells were then allowed to settle for 1 hour on microscopy slides and blocked with 2% BSA in PBS. To visualise the lysosome, cells were stained with mouse anti-p67 antibody. The antibodies were detected using goat anti-rabbit Alexa Fluor 488 or goat anti-mouse Alexa Fluor 594 antibodies, before analysis by immunofluorescence microscopy.

For the assays measuring uptake of biotinylated VSG, biotinylation of surface VSG and the subsequent VSG endocytosis assay was performed essentially as described in Engstler *et al* [20]. Cells (1 x 10^7^) were harvested and cooled to 4°C to block endocytosis. Cells were washed in TDB, and resuspended at a density of 1 x10^8^ cells/ ml, followed by a 10 minute incubation with 1 mM EZ-Link sulfo-NHS-SS-Biotin (Life Technologies) at 4°C. 10 mM Tris buffer (pH 7.5) was then added, and 1 x 10^7^ cells were transferred to pre-warmed HMI-9 medium with 10% FCS and incubated at 37°C to reinitiate endocytosis for 0, 0.5, 1, 1.5, 3, 6 or 12 minutes. Endocytosis was stopped by adding excess ice-cold stripping buffer (60% HMI-9, 10% FCS, 50 mM glutathione pH 9.0). Cells were washed in PBS/ 2% bovine serum albumin (BSA), fixed in 2% PFA and resuspended in 100 mM Na_2_HPO_4_/ 100 mM glycine pH 7.2 for 15 minutes. Cells were then permeabilised using 0.1% Triton X-100 and resuspended in 10 μg/ ml Streptavidin-Alexa Fluor 488 (Molecular Probes, Life Technologies) for one hour in the dark at room temperature. Cells were subsequently analysed by immunofluorescence microscopy or flow cytometry.

### Endocytosis assays

*T*. *brucei* 221VB1.2 cells (5 x 10^6^) were harvested per condition and cooled to 4°C to stop endocytosis. Cells were washed in TDB before being resuspended in TDB with the addition of either Dylight 488 labelled tomato lectin at 5 μg/ ml (Vector Labs), AlexaFluor 488 conjugated human transferrin at 50 μg/ ml (Life Technologies) or 10,000 MW AlexaFluor conjugated dextran at 5 mg/ ml (Life Technologies). Cells were subsequently transferred (without a wash step) to 37°C to re-activate endocytosis for 0, 0.5, 1, 2, 4, 8 or 16 minutes (in experiments following uptake of tomato lectin or transferrin) or 0, 1, 2, 4, 8, 16 or 32 minutes (in experiments following uptake of dextran). Cells were then washed in TDB, fixed in 2% PFA for 20 minutes and analysed by flow cytometry. Statistical analyses of VSG221 antibody clearance or uptake of the various endocytosis markers was performed by first fitting each data set to the non-linear regression model using the GraphPad Prism software. The half-time of uptake was then calculated for each data set and analysed using the Student’s t-test.

### Time-lapse live cell video microscopy

VSG synthesis was blocked in *T*. *brucei* 221VB1.2 cells by the induction of *VSG221* RNAi with tetracycline (1 μg/ ml) for eight hours prior to image capture. Cells (10 μl) in HMI-9 medium with the addition of 15% FCS were transferred into a chamber that was 0.1 mm deep (BIOPTECHS FCS2 & FCS3 closed chamber system), sealed with a coverslip, and allowed to equilibrate at 37°C for ten minutes. Cells were observed by bright-field light videomicroscopy using a Leica DM5500B microscope with a 10X NA 0.40 objective lens on a focal point midway through the sample depth. Each image was captured every 200 millisecond (5 Hz) for 512 frames (1.7 minutes) with an exposure time of 1 millisecond. Analysis of the videomicrographs was performed using custom designed macros on ImageJ [64]. Particles were identified using an intensity maxima algorithm. Tracks were constructed for each cell by finding the nearest particle to the expected cell location based on its motion in the previous two frames. Tracks were terminated if no particle was found within ten pixels (6.44 μm). Cell mean swimming speed, velocity and directional persistence were calculated from cell tracks as previously described [46]. Velocity is defined as total displacement over track length, while average speed is total distance travelled over track length. Statistical analyses on the mean swimming speed, velocity and directional persistence were performed using the Student’s t-test (n = 2500). μManager software was used for video capture [65].

## Supporting Information

S1 Fig Normal mouse serum is complement active. The effect of opsonisation on the viability of *T*. *brucei* after the induction of VSG RNAi. *VSG221* RNAi was induced in *T*. *brucei* 221VG1.1 for eight hours (h). Cells were either left untreated, or incubated with 10% normal mouse serum (NMS) from CD-1 mice, rabbit polyclonal anti-VSG221 antibody (1:5000 dilution) or both NMS and anti-VSG221 antibody. The number of live *T*. *brucei* was counted using a haemocytometer before and after the 90 minute incubation period. The number of live trypanosomes at 0 minutes is normalised to 100%, and the number after the 90 minute incubation is expressed as a relative percentage. Data show the average percentage (%) of viable trypanosomes from four independent biological replicates. Statistical analysis was performed using the Student’s t-test with the asterisk (*) indicating *P<0.05.
(TIF)

S2 Fig The anti-VSG221 antibody used was not at saturating concentrations. **(A)** Antibody saturation assay. *T*. *brucei* 221VB1.2 was cooled to 4°C to block endocytosis, and incubated with polyclonal rabbit anti-VSG221 antibody (1:5000 dilution) for one hour to coat the cells (1^st^ incubation). The trypanosomes were subsequently centrifuged, and the supernatant removed. The supernatant was subsequently incubated with new *T*. *brucei* (2^nd^ incubation). Cells retrieved after these two incubation steps were fixed in 2% PFA and incubated with the secondary goat anti-rabbit antibody coupled to Alexa Fluor 488. The amount of surface bound anti-VSG221 antibody was assessed using flow cytometry. **(B)** The amount of anti-VSG221 antibody present on the surface of cells which were incubated only with the primary anti-VSG221 antibody (1° Ab only), or the secondary goat anti-VSG221 antibody (2° Ab only) or using both antibodies after the first incubation. Anti-VSG221 antibody levels were also determined on fresh *T*. *brucei* after incubation with the supernatant (2^nd^ incubation). These experiments were performed in cells in the presence or absence of VSG221 RNAi for eight hours (h).
(TIF)

S3 Fig Blocking synthesis of the essential chromatin protein TDP1 does not result in a decrease in clearance of surface-bound anti-VSG221 antibodies. **(A)** Surface clearance of anti-VSG221 antibodies was measured using flow cytometry in *T*. *brucei* 90–13 TDPC1 cells where *TDP1* RNAi was induced for 0 or 24 hours (h). The cells were next transferred to 4°C to arrest endocytosis, and then opsonised with anti-VSG221 antibodies. Cells were subsequently transferred to 37°C to reinitiate endocytosis for the time indicated in minutes. The reaction was then stopped, the cells were fixed and stained with secondary antibody coupled to Alexa Fluor 488 and propidium iodide to stain DNA. The amount of surface-bound anti-VSG221 antibody was determined at the G1 (orange), S phase (blue) or G2/ M (purple) cell cycle stages. **(B)** Quantitation of the reduced clearance of anti-VSG221 antibody after blocking TDP1 synthesis. Mean fluorescence intensities are shown as a percentage (%) of the value at 0 minutes. The results that are shown are the mean of three independent biological replicates with the standard deviation indicated with error bars. **(C)** Quantitation of the half-life of anti-VSG221 antibodies after blocking TDP1 synthesis. Results shown are the mean of three independent biological replicates with the standard deviation indicated with error bars. After fitting each data set to the non-linear regression model, statistical analysis was performed using the Student’s t-test.
(TIF)

S4 Fig Quantitation of VSG221 after the induction of *VSG221* RNAi. **(A)** Quantitation of total VSG221 in *T*. *brucei* after the induction of *VSG221* RNAi for eight hours, using LiCor analysis of protein lysates. Two fold dilutions of cell extract containing 2 x 10^4^ cells per well are analysed. The *T*. *brucei* SM221pur cell line expresses VSG221 and *T*. *brucei* HN1(V02+) expresses VSGV02 [66]. The BF *T*. *brucei* 221VG1.1 was analysed before or after the induction of *VSG221* RNAi for eight hours. The Hsp70 protein was used as a loading control [40]. **(B)** Quantitation of total VSG221 normalised to Hsp70. **(C)** The amount of VSG present on the *T*. *brucei* surface after the induction of VSG RNAi for 8 hours as assessed using biotinylation. *T*. *brucei* 221VB1.2 cells were cooled to 4°C to stop endocytosis, and then biotinylated using 1 mM biotin [20]. Cells were fixed, and biotinylation of surface VSG was detected using Alexa488-conjugated streptavidin and quantitated by flow cytometry. *T*. *brucei* DNA was simultaneously stained with propidium iodide and cells were analysed according to respective cell cycle stage: G1, S and G2-M. The overall change in biotinylation of the whole population is indicated under “total”. Results shown are the mean of three independent biological replicates with the standard deviation indicated with error bars. Statistical analysis was performed using the Student’s t-test *P<0.05. **(D)** Quantification of anti-VSG221 antibody present on the trypanosome cell surface in the presence or absence of *VSG221* RNAi for 8 hours. Cells were incubated with anti-VSG221 antibody at 4°C. Surface antibody binding was detected using an Alexa 488-conjugated secondary antibody, and total fluorescence was quantitated by flow cytometry. Results shown are the mean of three independent biological replicates with the standard deviation indicated with error bars. Statistical analysis was performed using the Student’s t-test *P<0.05.
(TIF)

S5 Fig The average swim speed is not significantly affected by blocking VSG221 synthesis for 8 hours (h). Either the whole cell population (pop.) or bi-flagellated cells were analysed. Bi-flagellated cells were selected manually from the videos of three independent biological replicates. 1500 tracks from the whole cell population or 50 tracks from bi-flagellated cells were analysed. The swim speed is shown in μm per second (s). Statistical significance was determined with Student’s t-test (***P<0.0001).
(TIF)
