# Supplementary figures and images for: Blocking Synthesis of the Variant Surface Glycoprotein Coat in Trypanosoma brucei Leads to an Increase in Macrophage Phagocytosis Due to Reduced Clearance of Surface Coat Antibodies
Source: PLoS Pathog. 2016 Nov 28;12(11):e1006023. doi: 10.1371/journal.ppat.1006023 (PMC5125712; doi:10.1371/journal.ppat.1006023)

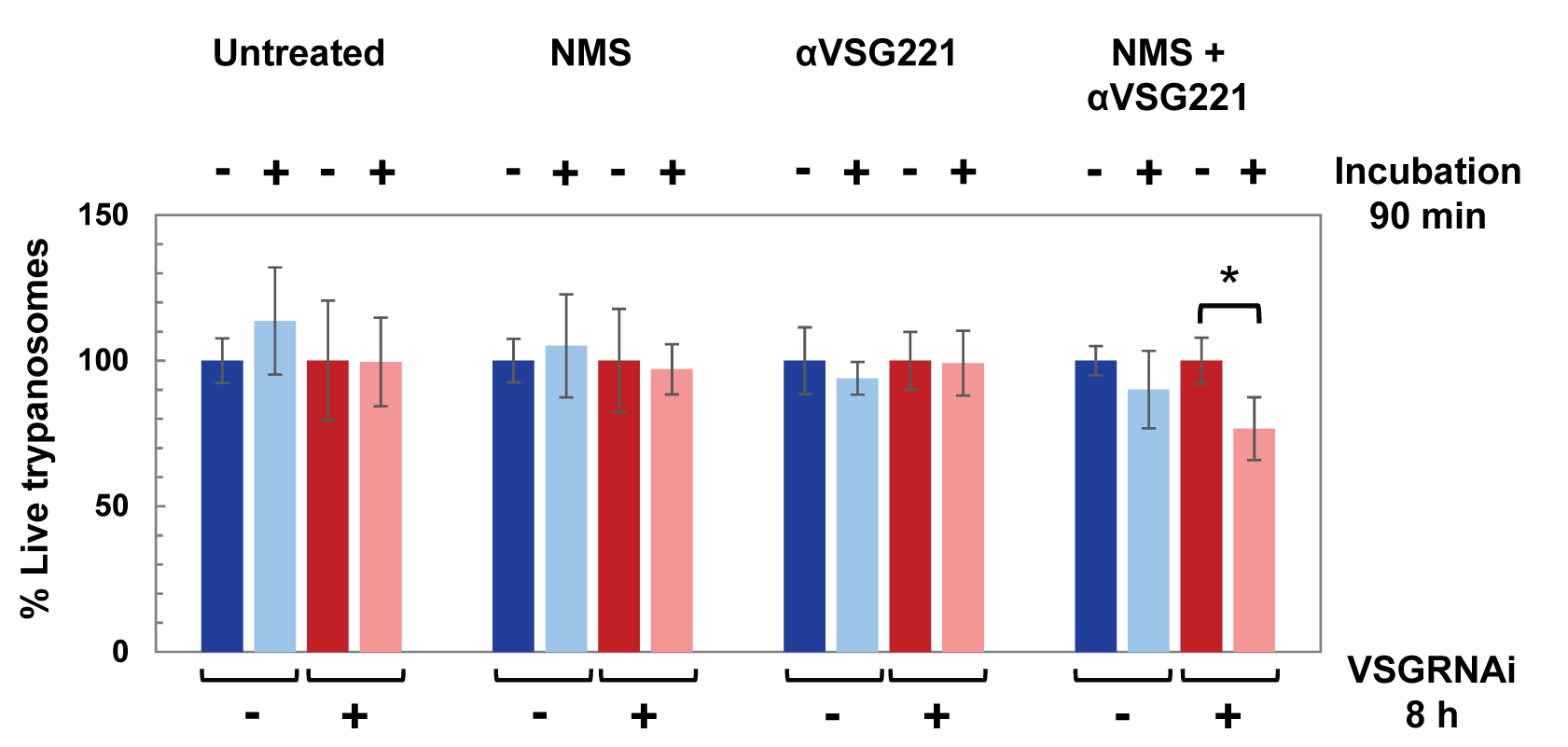

Supplement: S1 Fig — The effect of opsonisation on the viability of T. brucei after the induction of VSG RNAi. VSG221 RNAi was induced in T. brucei 221VG1.1 for eight hours (h). Cells were either left untreated, or incubated with 10% normal mouse serum (NMS) from CD-1 mice, rabbit polyclonal anti-VSG221 antibody (1:5000 dilution) or both NMS and anti-VSG221 antibody. The number of live T. brucei was counted using a haemocytometer before and after the 90 minute incubation period. The number of live trypanosomes at 0 minutes is normalised to 100%, and the number after the 90 minute incubation is expressed as a relative percentage. Data show the average percentage (%) of viable trypanosomes from four independent biological replicates. Statistical analysis was performed using the Student’s t-test with the asterisk (*) indicating *P<0.05. (TIF) [file ppat.1006023.s001.tif]

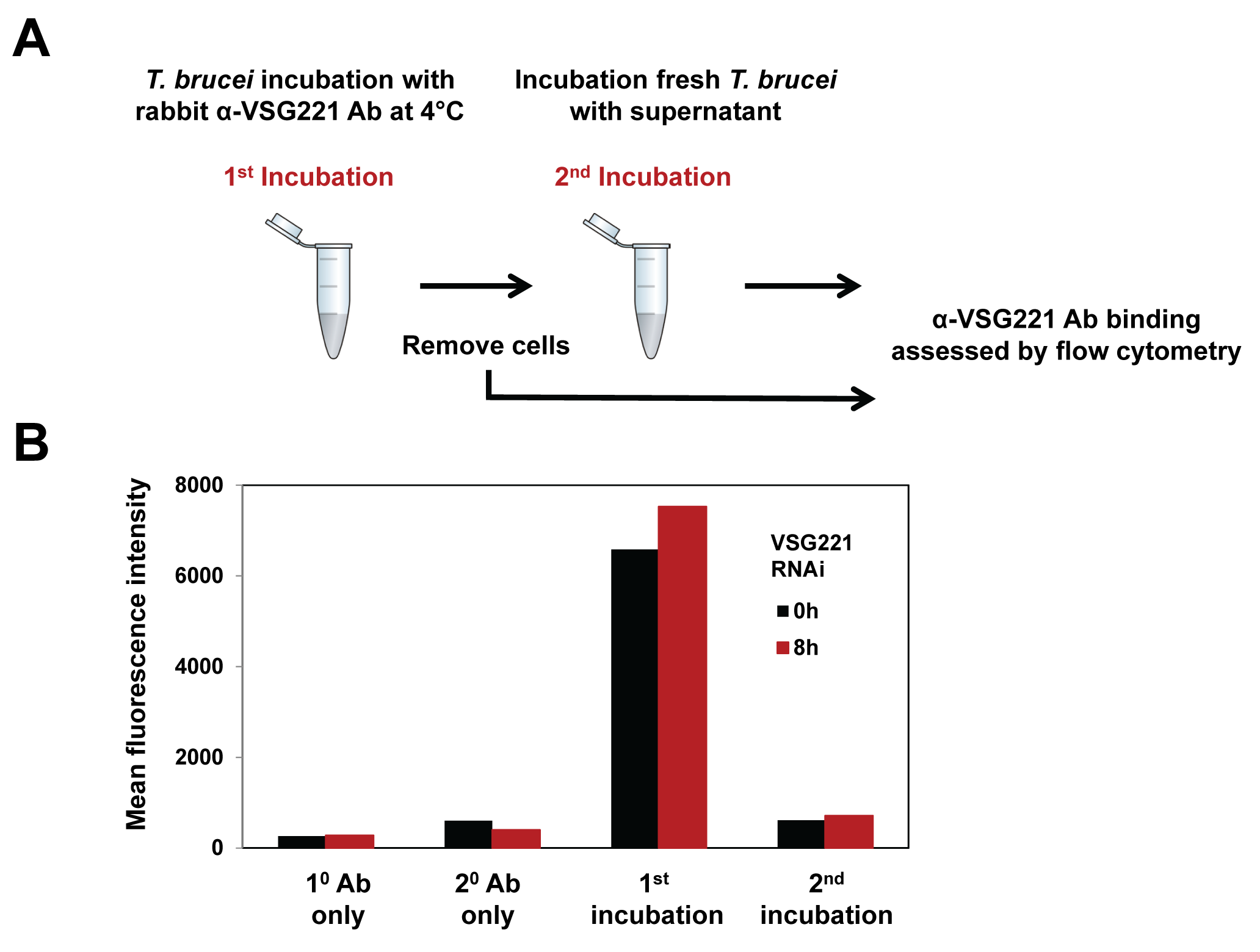

Supplement: S2 Fig — (A) Antibody saturation assay. T. brucei 221VB1.2 was cooled to 4°C to block endocytosis, and incubated with polyclonal rabbit anti-VSG221 antibody (1:5000 dilution) for one hour to coat the cells (1st incubation). The trypanosomes were subsequently centrifuged, and the supernatant removed. The supernatant was subsequently incubated with new T. brucei (2nd incubation). Cells retrieved after these two incubation steps were fixed in 2% PFA and incubated with the secondary goat anti-rabbit antibody coupled to Alexa Fluor 488. The amount of surface bound anti-VSG221 antibody was assessed using flow cytometry. (B) The amount of anti-VSG221 antibody present on the surface of cells which were incubated only with the primary anti-VSG221 antibody (1° Ab only), or the secondary goat anti-VSG221 antibody (2° Ab only) or using both antibodies after the first incubation. Anti-VSG221 antibody levels were also determined on fresh T. brucei after incubation with the supernatant (2nd incubation). These experiments were performed in cells in the presence or absence of VSG221 RNAi for eight hours (h). (TIF) [file ppat.1006023.s002.tif]

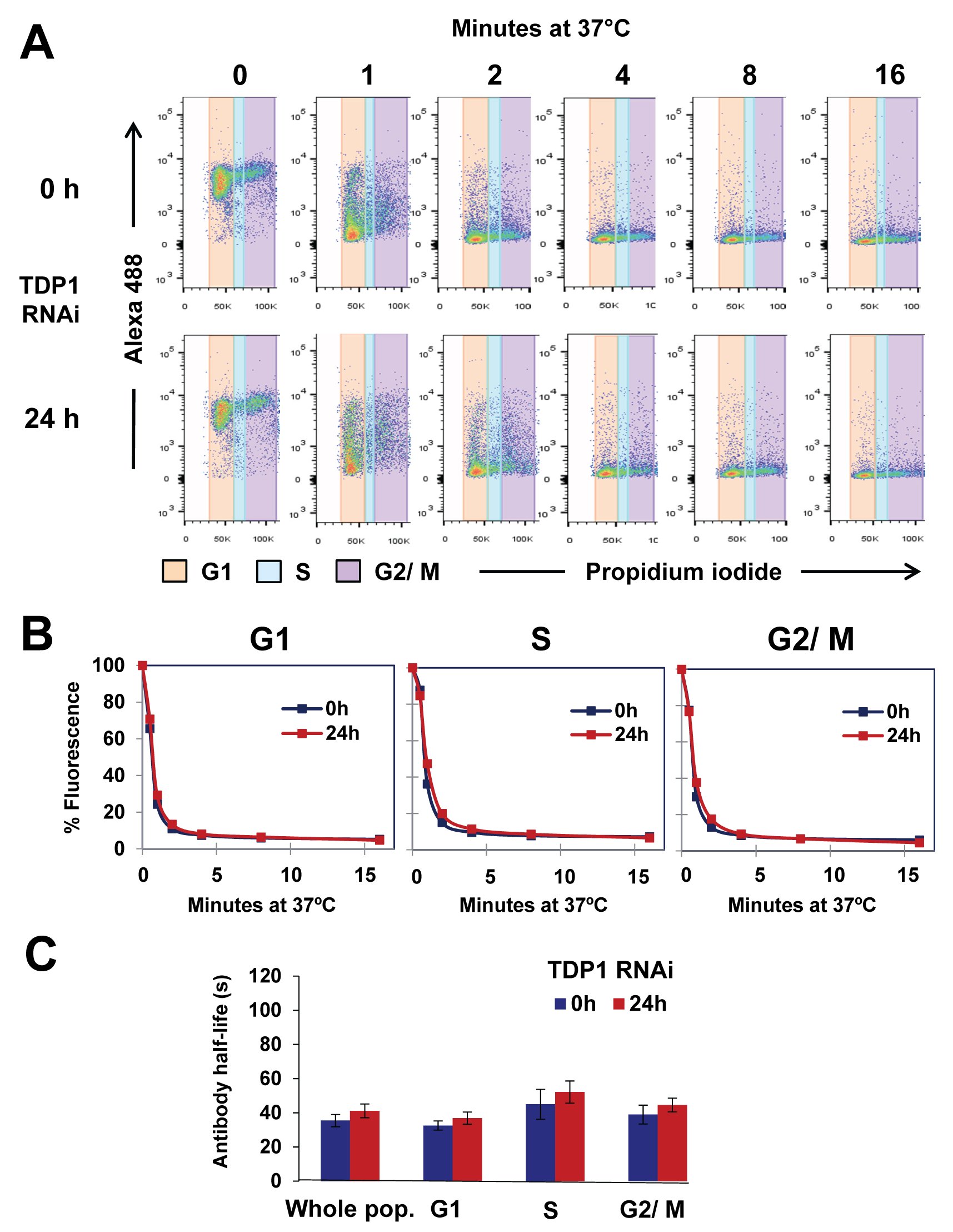

Supplement: S3 Fig — (A) Surface clearance of anti-VSG221 antibodies was measured using flow cytometry in T. brucei 90–13 TDPC1 cells where TDP1 RNAi was induced for 0 or 24 hours (h). The cells were next transferred to 4°C to arrest endocytosis, and then opsonised with anti-VSG221 antibodies. Cells were subsequently transferred to 37°C to reinitiate endocytosis for the time indicated in minutes. The reaction was then stopped, the cells were fixed and stained with secondary antibody coupled to Alexa Fluor 488 and propidium iodide to stain DNA. The amount of surface-bound anti-VSG221 antibody was determined at the G1 (orange), S phase (blue) or G2/ M (purple) cell cycle stages. (B) Quantitation of the reduced clearance of anti-VSG221 antibody after blocking TDP1 synthesis. Mean fluorescence intensities are shown as a percentage (%) of the value at 0 minutes. The results that are shown are the mean of three independent biological replicates with the standard deviation indicated with error bars. (C) Quantitation of the half-life of anti-VSG221 antibodies after blocking TDP1 synthesis. Results shown are the mean of three independent biological replicates with the standard deviation indicated with error bars. After fitting each data set to the non-linear regression model, statistical analysis was performed using the Student’s t-test. (TIF) [file ppat.1006023.s003.tif]

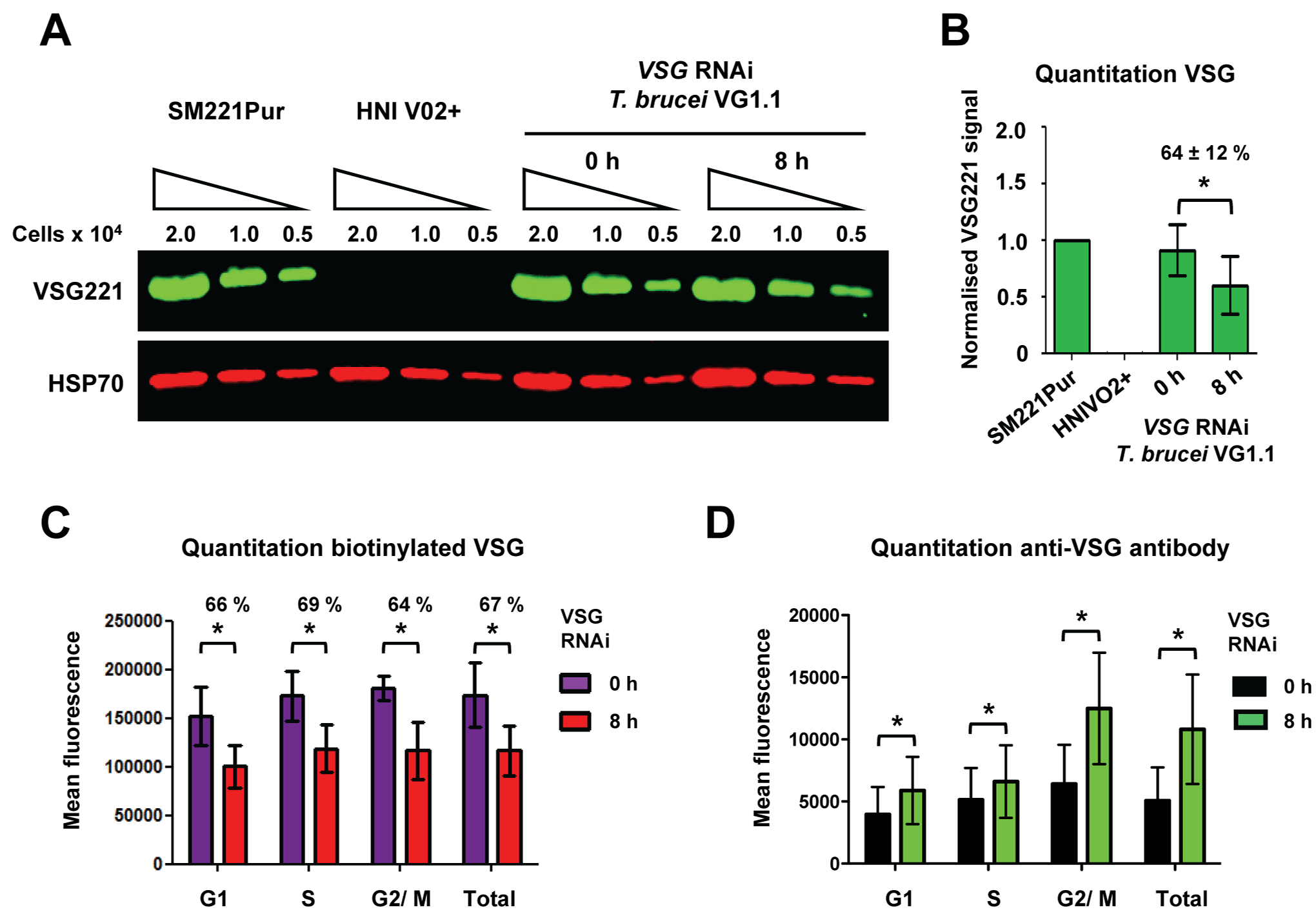

Supplement: S4 Fig — (A) Quantitation of total VSG221 in T. brucei after the induction of VSG221 RNAi for eight hours, using LiCor analysis of protein lysates. Two fold dilutions of cell extract containing 2 x 10^4^ cells per well are analysed. The T. brucei SM221pur cell line expresses VSG221 and T. brucei HN1(V02+) expresses VSGV02 [66]. The BF T. brucei 221VG1.1 was analysed before or after the induction of VSG221 RNAi for eight hours. The Hsp70 protein was used as a loading control [40]. (B) Quantitation of total VSG221 normalised to Hsp70. (C) The amount of VSG present on the T. brucei surface after the induction of VSG RNAi for 8 hours as assessed using biotinylation. T. brucei 221VB1.2 cells were cooled to 4°C to stop endocytosis, and then biotinylated using 1 mM biotin [20]. Cells were fixed, and biotinylation of surface VSG was detected using Alexa488-conjugated streptavidin and quantitated by flow cytometry. T. brucei DNA was simultaneously stained with propidium iodide and cells were analysed according to respective cell cycle stage: G1, S and G2-M. The overall change in biotinylation of the whole population is indicated under “total”. Results shown are the mean of three independent biological replicates with the standard deviation indicated with error bars. Statistical analysis was performed using the Student’s t-test *P<0.05. (D) Quantification of anti-VSG221 antibody present on the trypanosome cell surface in the presence or absence of VSG221 RNAi for 8 hours. Cells were incubated with anti-VSG221 antibody at 4°C. Surface antibody binding was detected using an Alexa 488-conjugated secondary antibody, and total fluorescence was quantitated by flow cytometry. Results shown are the mean of three independent biological replicates with the standard deviation indicated with error bars. Statistical analysis was performed using the Student’s t-test *P<0.05. (TIF) [file ppat.1006023.s004.tif]

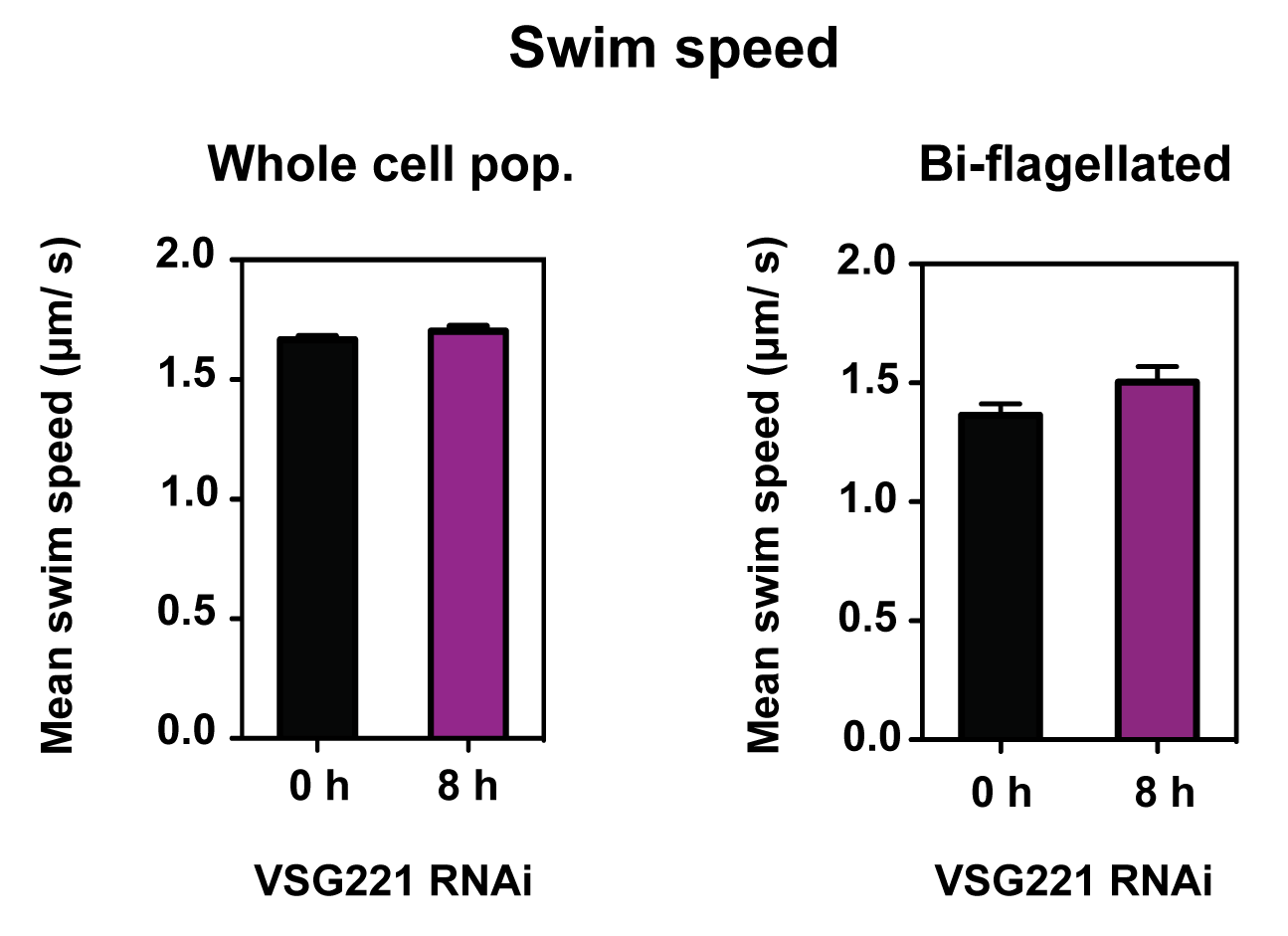

Supplement: S5 Fig — Either the whole cell population (pop.) or bi-flagellated cells were analysed. Bi-flagellated cells were selected manually from the videos of three independent biological replicates. 1500 tracks from the whole cell population or 50 tracks from bi-flagellated cells were analysed. The swim speed is shown in μm per second (s). Statistical significance was determined with Student’s t-test (***P<0.0001). (TIF) [file ppat.1006023.s005.tif]
